# Supplementary material for: DPHL v.2: An updated and comprehensive DIA pan-human assay library for quantifying more than 14,000 proteins
Source: Patterns (N Y). 2023 Jul 5;4(7):100792. doi: 10.1016/j.patter.2023.100792 (PMC10382975; doi:10.1016/j.patter.2023.100792)
Supplement: Document 1. Figures S1–S10 [file mmc1.pdf]

Patterns, Volume *4*

Supplemental information

DPHL v.2: An updated and comprehensive DIA pan-human assay library for quantifying more than 14,000 proteins

Zhangzhi Xue, Tiansheng Zhu, Fangfei Zhang, Cheng Zhang, Nan Xiang, Liujia Qian, Xiao Yi, Yaoting Sun, Wei Liu, Xue Cai, Linyan Wang, Xizhe Dai, Liang Yue, Lu Li, Thang V. Pham, Sander R. Piersma, Qi Xiao, Meng Luo, Cong Lu, Jiang Zhu, Yongfu Zhao, Guangzhi Wang, Junhong Xiao, Tong Liu, Zhiyu Liu, Yi He, Qijun Wu, Tingting Gong, Jianqin Zhu, Zhiguo Zheng, Juan Ye, Yan Li, Connie R. Jimenez, Jun A, and Tiannan Guo

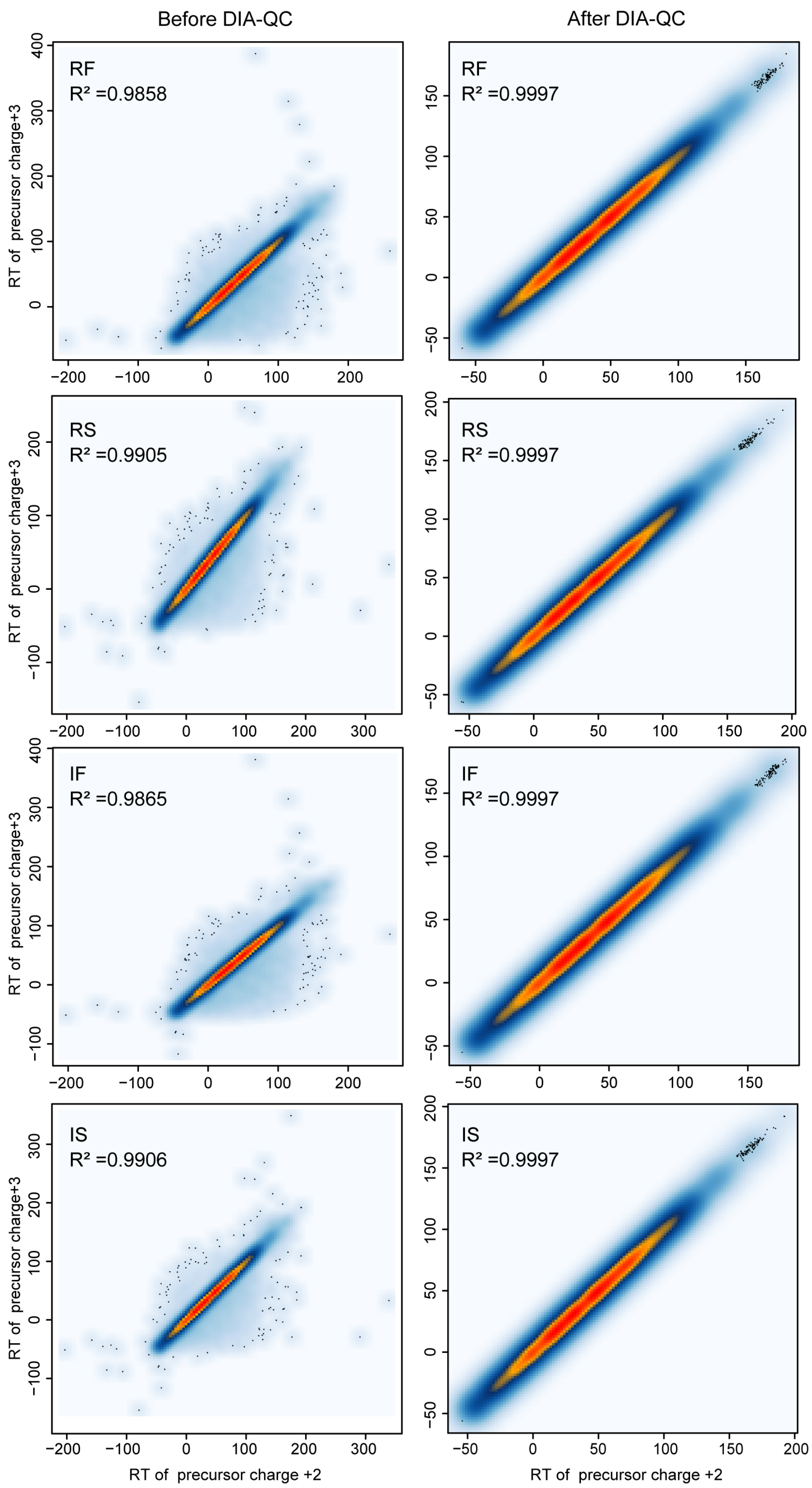

Figure S1. Correlation of paired peptides with RT correction values before and after QC.

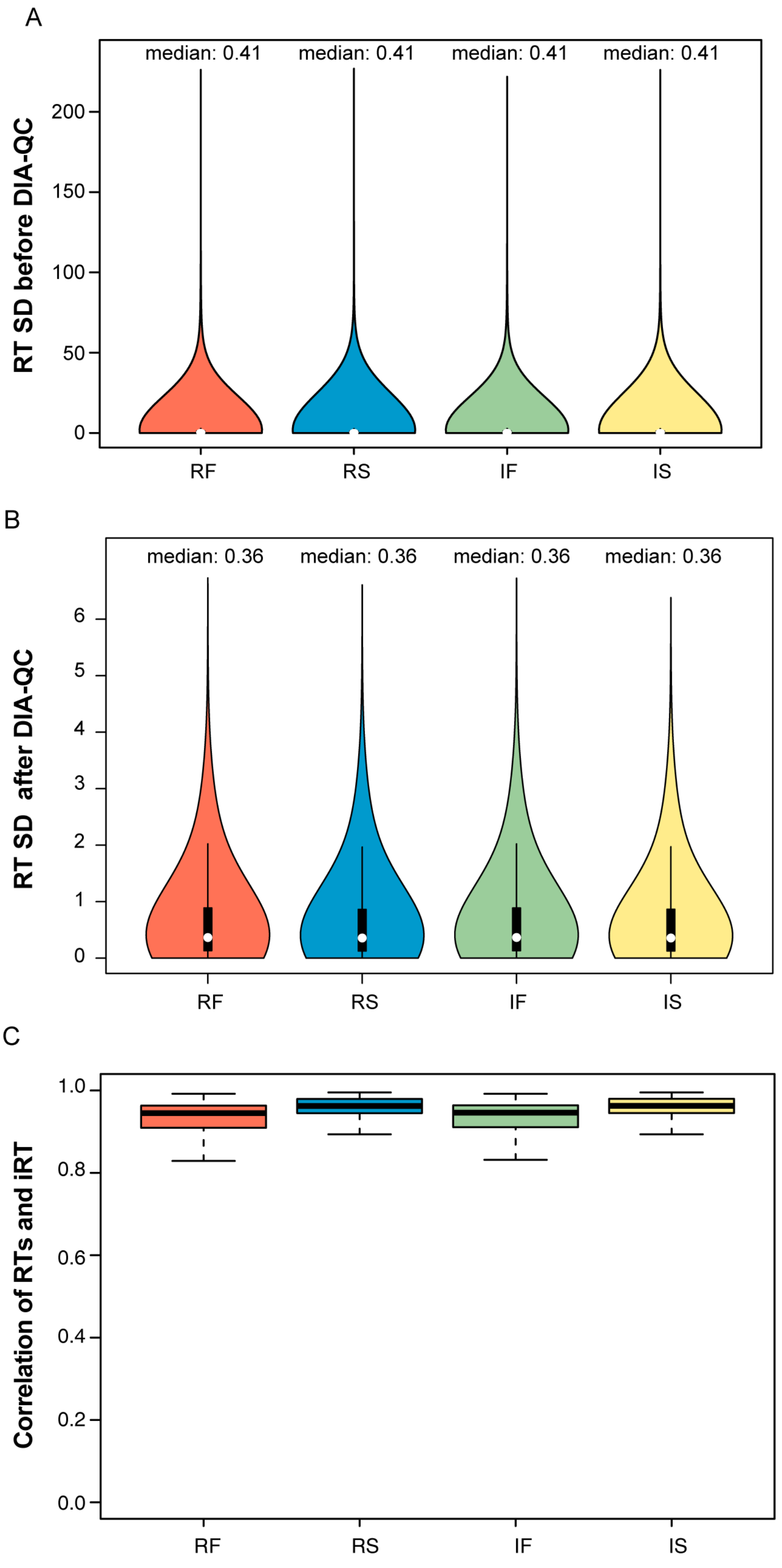

Figure S2. Standard deviation (SD) of paired peptides with RT values before (A) and after (B) QC. (C) The correlation between the RTs and iRT values in each sample of the four libraries.

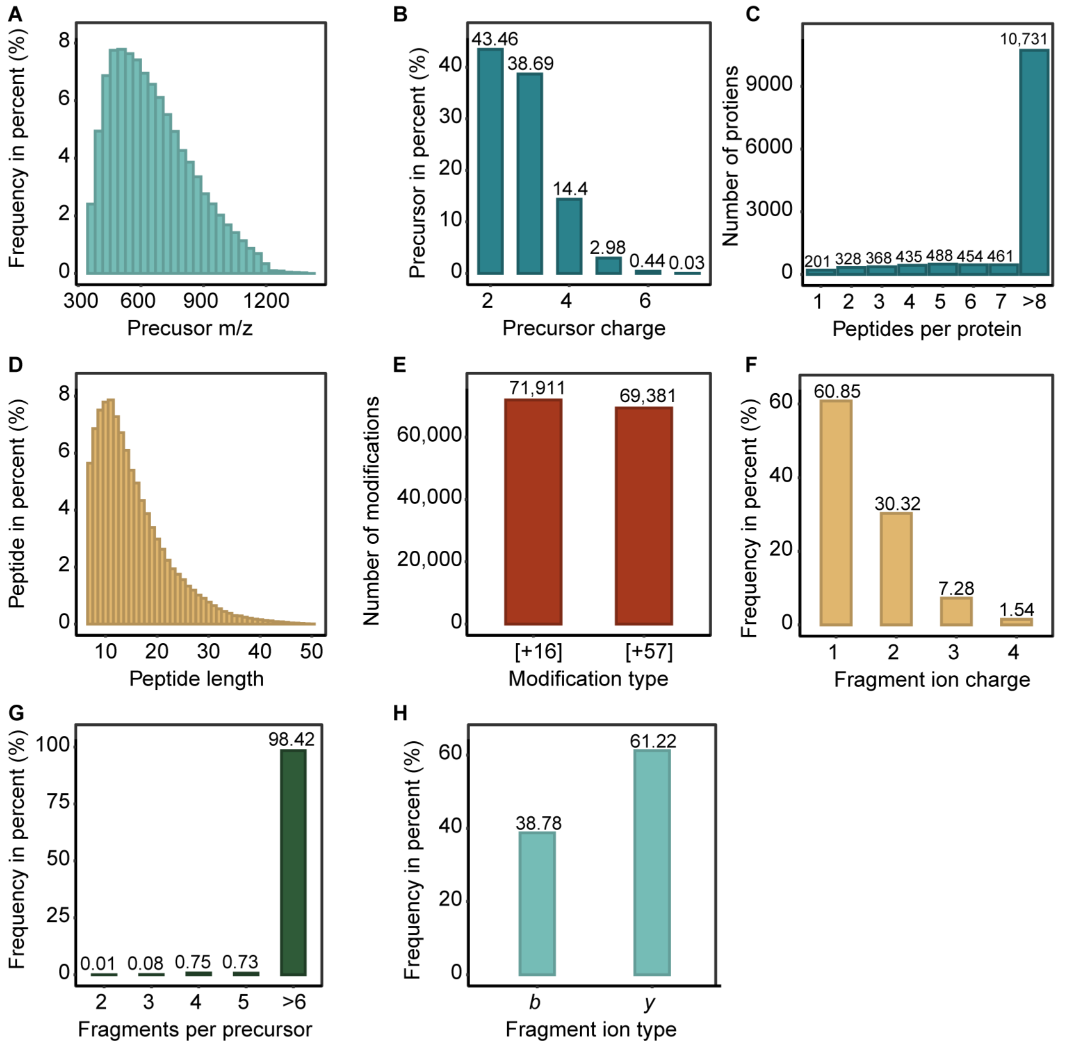

Figure S3. Characteristics of the RF library. (A) Distribution of precursors' m/z. (B) Counts of different precursor charge states. (C) Number of proteotypic peptides for each protein. (D) Distribution of peptide lengths. (E) Number of peptides with either of two modifications (+16: oxidation in methionine; +57: carbamidomethylation in cysteine). (F) The proportion of different charges of fragment ions. (G) Proportion of fragment ions per precursor ion. (H) Percentage of b and y ions.

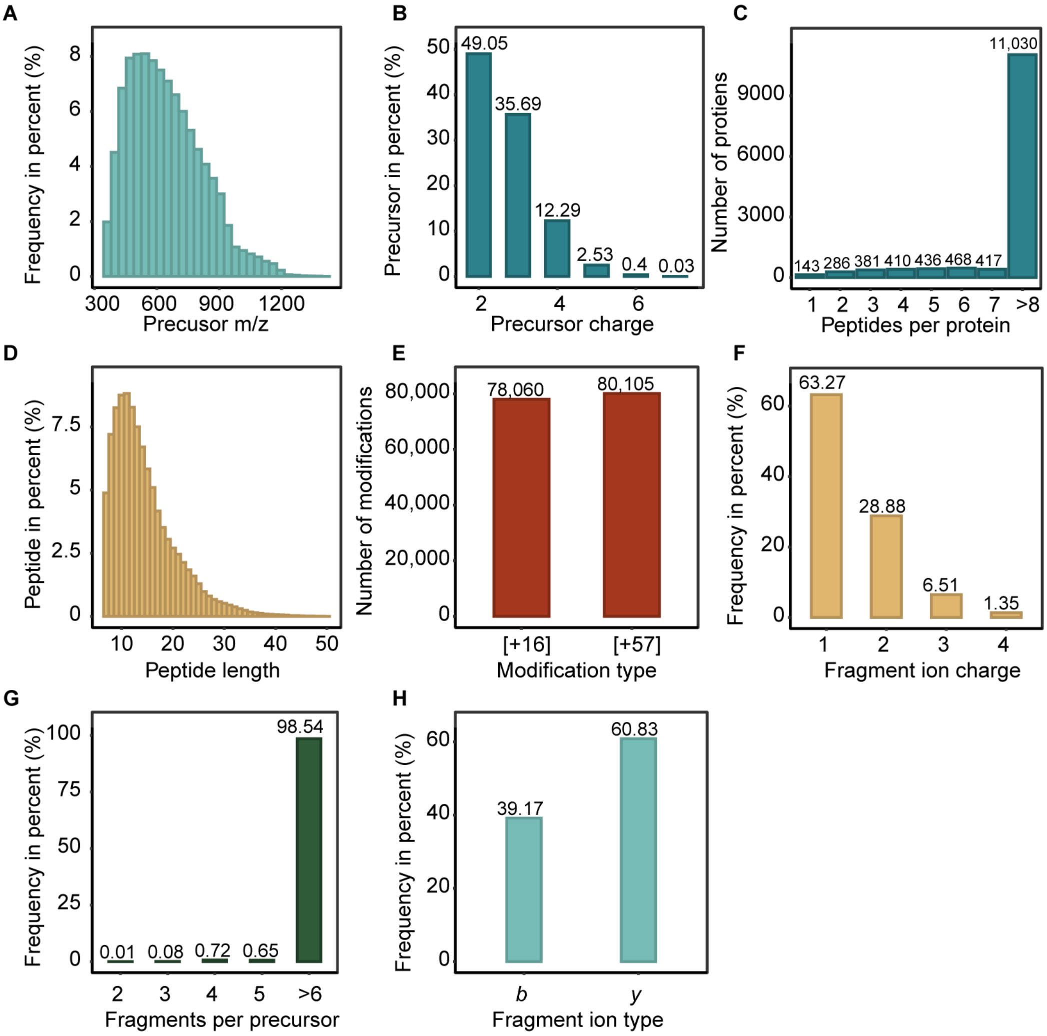

Figure S4. Characteristics of the RS library. (A) Distribution of precursors' m/z. (B) Counts of different precursor charge states. (C) Number of proteotypic peptides for each protein. (D) Distribution of peptide lengths. (E) Number of peptides with either of two modifications (+16: oxidation in methionine; +57: carbamidomethylation in cysteine). (F) The proportion of different charges of fragment ions. (G) Proportion of fragment ions per precursor ion. (H) Percentage of b and y ions.

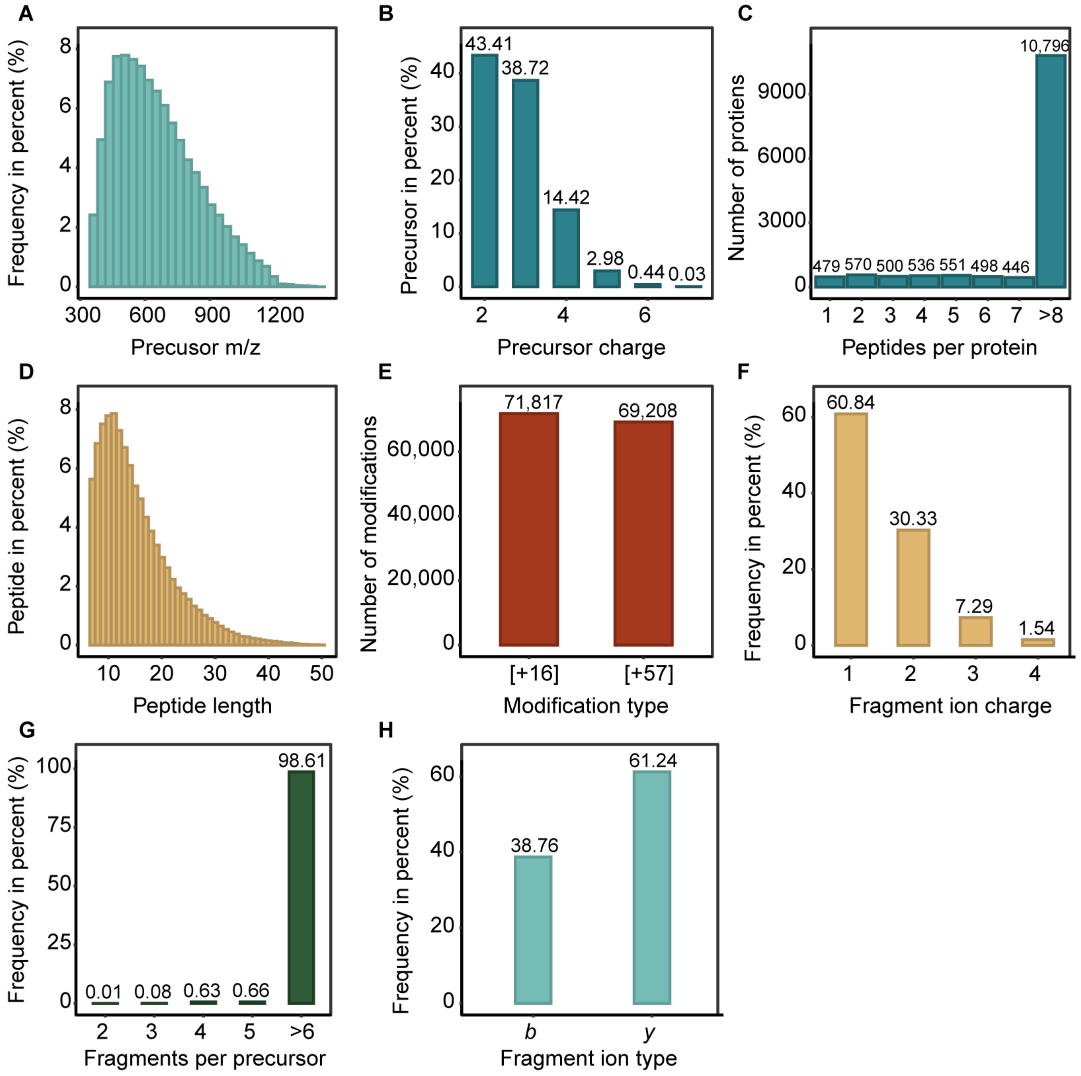

Figure S5. Characteristics of the IF library. (A) Distribution of precursors' m/z. (B) Counts of different precursor charge states. (C) Number of proteotypic peptides for each protein. (D) Distribution of peptide lengths. (E) Number of peptides with either of two modifications (+16: oxidation in methionine; +57: carbamidomethylation in cysteine). (F) The proportion of different charges of fragment ions. (G) Proportion of fragment ions per precursor ion. (H) Percentage of b and y ions.

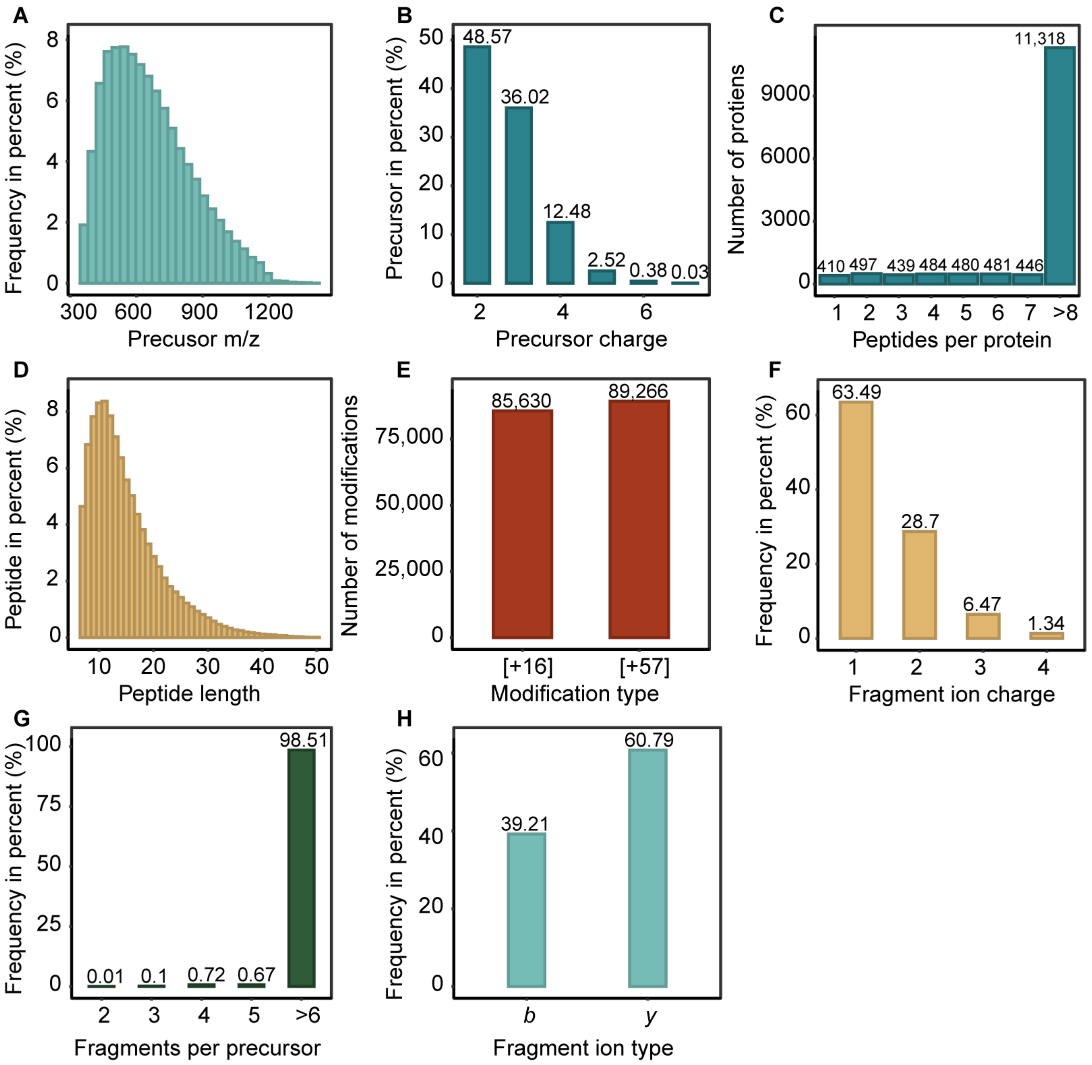

Figure S6. Characteristics of the IS library. (A) Distribution of precursors' m/z. (B) Counts of different precursor charge states. (C) Number of proteotypic peptides for each protein. (D) Distribution of peptide lengths. (E) Number of peptides with either of two modifications (+16: oxidation in methionine; +57: carbamidomethylation in cysteine). (F) The proportion of different charges of fragment ions. (G) Proportion of fragment ions per precursor ion. (H) Percentage of b and y ions.

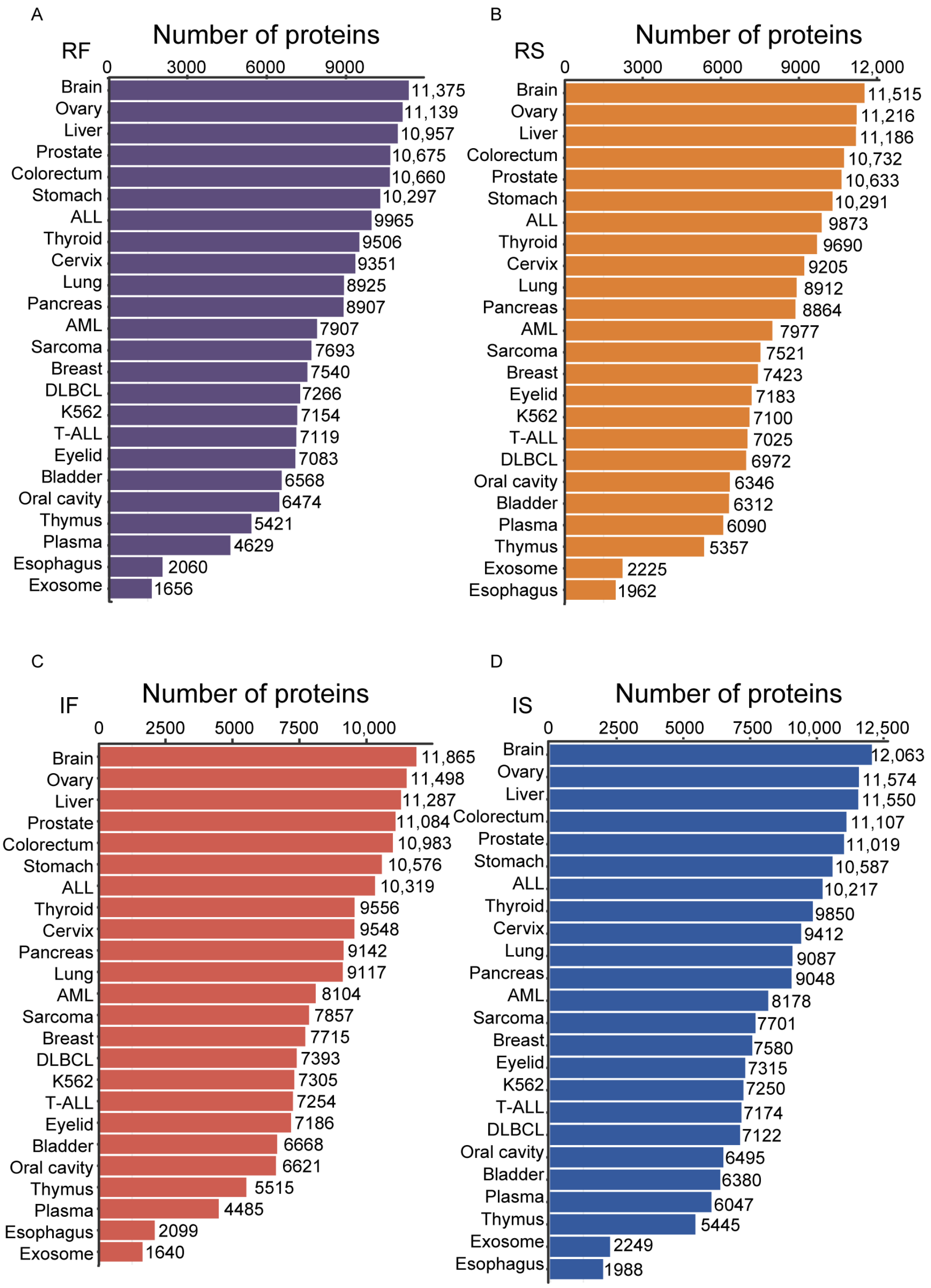

Figure S7. Bar plots displaying the number of identified proteins using our four libraries for each sample type. RF, reviewed fasta sequence & full-specific digestion mode; RS, reviewed fasta sequence & semi-specific digestion mode; IF, isoform fasta sequence & full-specific digestion mode; IS, isoform fasta sequence & semi-specific digestion mode; ALL, acute lymphoblastic leukemia.

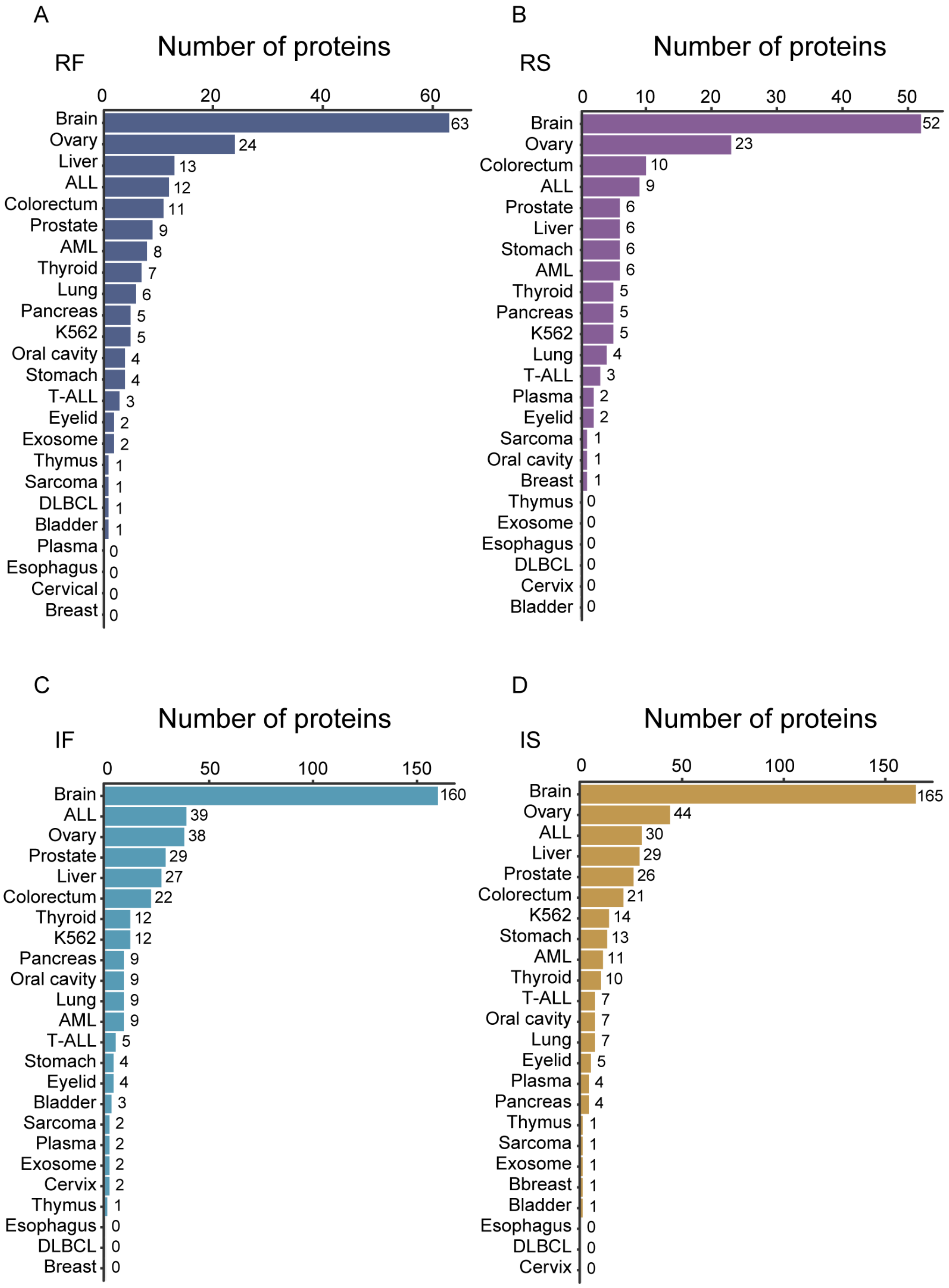

Figure S8. Bar plots displaying the number of identified unique proteins for each sample type. RF, reviewed fasta sequence & full-specific digestion mode; RS, reviewed fasta sequence & semi-specific digestion mode; IF, isoform fasta sequence & full-specific digestion mode; IS, isoform fasta sequence & semi-specific digestion mode; ALL, acute lymphoblastic leukemia.

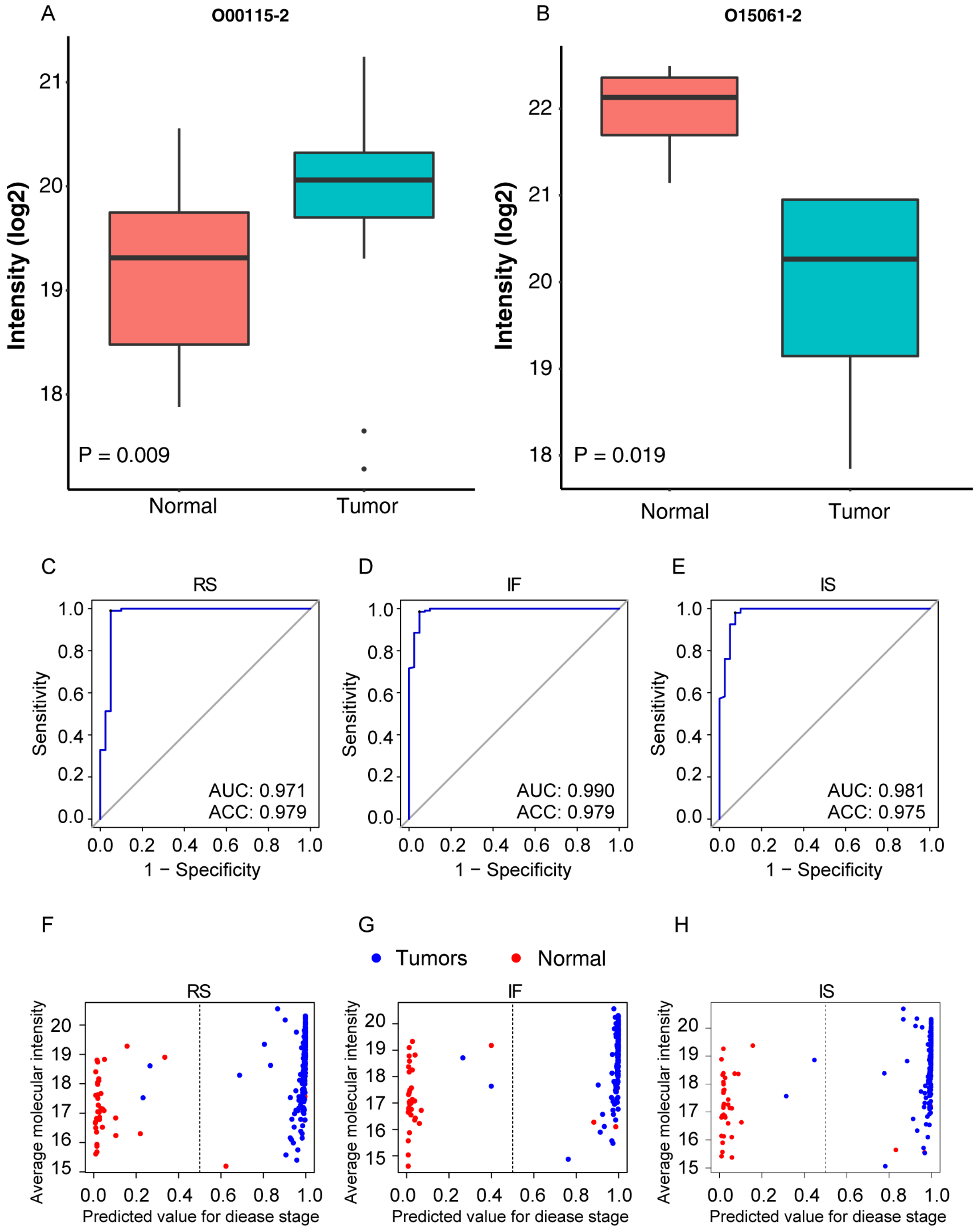

Figure S9. (A-B) The expression levels of the O00115-2 and the O15061-2. (C-E) ROC plots of the RS set, the IF set, and the IS set. (F-H) Performance of the model in the RS set, the IF set, and the IS set. RF, reviewed fasta sequence & full-specific digestion mode; RS, reviewed fasta sequence & semi-specific digestion mode; IF, isoform fasta sequence & full-specific digestion mode; IS, isoform fasta sequence & semi-specific digestion mode.

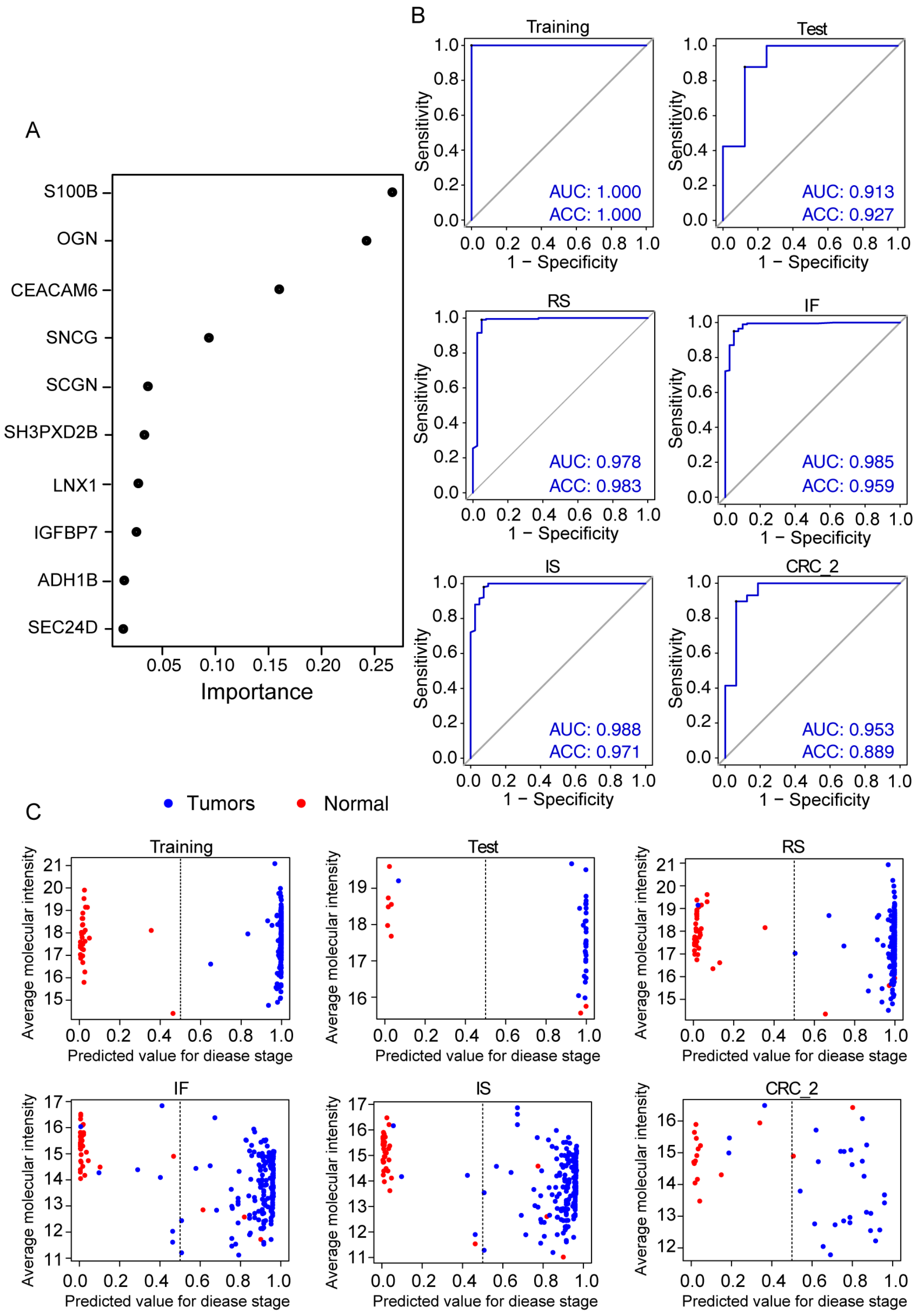

Figure S10. Machine learning to identify potential CRC biomarkers (using all differentially expressed proteins from the RF set). (A) Prioritization of 10 important variables. (B) Performance of the model in the training set, the test set, the RS set, the IF set, the IS set, and the CRC_2 dataset. RF, reviewed fasta sequence & full-specific digestion mode; RS, reviewed fasta sequence & semi-specific digestion mode; IF, isoform fasta sequence & full-specific digestion mode; IS, isoform fasta sequence & semi-specific digestion mode.
