# Supplementary material for: DPHL v.2: An updated and comprehensive DIA pan-human assay library for quantifying more than 14,000 proteins
Source: Patterns (N Y). 2023 Jul 5;4(7):100792. doi: 10.1016/j.patter.2023.100792 (PMC10382975; doi:10.1016/j.patter.2023.100792)
Supplement: Document S2. Article plus supplemental information [file mmc5.pdf]

Descriptor

Patterns

DPHL v.2: An updated and comprehensive DIA pan-human assay library for quantifying more than 14,000 proteins

Highlights

- DPHL v.2 provides spectral information for 14,000+ proteins from 24 human tissue types
- Four library variants including protein isoforms and semi-tryptic peptides
- Highest number of proteins identified in the brain
- Inclusion of 452 FDA-approved drug targets and 100 ovary-enriched proteins

Authors

Zhangzhi Xue, Tiansheng Zhu, Fangfei Zhang, ..., Connie R. Jimenez, Jun A, Tiannan Guo

Correspondence

c.jimenez@amsterdamumc.nl (C.R.J.), ajun@westlake.edu.cn (J.A.), guotiannan@westlake.edu.cn (T.G.)

In brief

Interpretation of data-independent acquisition (DIA) mass spectrometry is facilitated by *a priori* knowledge of spectral libraries. Here, we present a comprehensive pan-human spectral library (DPHL v.2) that enables targeted proteomic analysis of over 14,000 proteins from 24 human tissue types, including semi-tryptic peptides and protein isoforms. The effectiveness of DPHL v.2 in facilitating biomarker discovery has been demonstrated using two colorectal cancer cohorts.

Xue et al., 2023, Patterns *4*, 100792
July 14, 2023 © 2023 The Author(s).
https://doi.org/10.1016/j.patter.2023.100792

CellPress

Patterns

CellPress OPEN ACCESS

Descriptor

DPHL v.2: An updated and comprehensive DIA pan-human assay library for quantifying more than 14,000 proteins

Zhangzhi Xue,[1,2,3] Tiansheng Zhu,[1,2,3,15] Fangfei Zhang,[1,2,3] Cheng Zhang,[1,2,3] Nan Xiang,[4] Liujia Qian,[1,2,3] Xiao Yi,[4] Yaoting Sun,[1,2,3] Wei Liu,[4] Xue Cai,[1,2,3] Linyan Wang,[5] Xizhe Dai,[5] Liang Yue,[1,2,3] Lu Li,[1,2,3] Thang V. Pham,[6] Sander R. Piersma,[6] Qi Xiao,[1,2,3] Meng Luo,[7] Cong Lu,[8] Jiang Zhu,[8] Yongfu Zhao,[9] Guangzhi Wang,[9] Junhong Xiao,[9] Tong Liu,[10] Zhiyu Liu,[11] Yi He,[11] Qijun Wu,[12] Tingting Gong,[12] Jianqin Zhu,[13,14] Zhiguo Zheng,[13,14] Juan Ye,[5] Yan Li,[7] Connie R. Jimenez,[6,*] Jun A,[1,2,3,*] and Tiannan Guo[1,2,3,16,*]

[1]iMarker Lab, Westlake Laboratory of Life Sciences and Biomedicine, Key Laboratory of Structural Biology of Zhejiang Province, School of Life Sciences, Westlake University, Hangzhou, Zhejiang Province 310024, China

[2]Institute of Basic Medical Sciences, Westlake Institute for Advanced Study, Hangzhou, Zhejiang Province 310024, China

[3]Research Center for Industries of the Future, Westlake University, 600 Dunyu Road, Hangzhou, Zhejiang 310030, China

[4]Westlake Omics (Hangzhou) Biotechnology Co., Ltd., Hangzhou 310024, China

[5]Department of Ophthalmology, The Second Affiliated Hospital, Zhejiang University School of Medicine, Hangzhou, Zhejiang 310000, China

[6]OncoProteomics Laboratory, Department of Medical Oncology, VU University Medical Center, VU University, 1011 Amsterdam, the Netherlands

(Affiliations continued on next page)

THE BIGGER PICTURE With thousands of proteins, the human proteome is a complex system that can be analyzed through mass spectrometry (MS)-based proteomics. In this method, proteins are usually broken down into peptides at specific sites, which are then separated using liquid chromatography (LC) and ionized. This process produces ionized peptides that can be comprehensively digitized with data-independent acquisition (DIA)-MS. However, interpreting DIA-MS data requires spectral libraries that document the retention time and intensities of peptide precursors and fragment ions. Here, we present the most comprehensive spectral library, DPHL v.2, for the human proteome. The spectral library is built from 1,608 data-dependent acquisition (DDA)-MS data obtained from 24 human tissue types, providing spectral information for over 14,000 proteins. It can also be used for developing targeted proteomics assays. This resource potentially facilitates disease diagnosis and accelerates drug discovery.

1 2 3 4 5 Mainstream: Data science output is well understood and (nearly) universally adopted

SUMMARY

A comprehensive pan-human spectral library is critical for biomarker discovery using mass spectrometry (MS)-based proteomics. DPHL v.1, a previous pan-human library built from 1,096 data-dependent acquisition (DDA) MS data of 16 human tissue types, allows quantifying of 10,943 proteins. Here, we generated DPHL v.2 from 1,608 DDA-MS data. The data included 586 DDA-MS data acquired from 18 tissue types, while 1,022 files were derived from DPHL v.1. DPHL v.2 thus comprises data from 24 sample types, including several cancer types (lung, breast, kidney, and prostate cancer, among others). We generated four variants of DPHL v.2 to include semi-tryptic peptides and protein isoforms. DPHL v.2 was then applied to two colorectal cancer cohorts. The numbers of identified and significantly dysregulated proteins increased by at least 21.7% and 14.2%, respectively, compared with DPHL v.1. Our findings show that the increased human proteome coverage of DPHL v.2 provides larger pools of potential protein biomarkers.

INTRODUCTION

Mass spectrometry (MS)-based quantitative proteomics is widely used for protein biomarker discovery.[1–3] Subsequent biomarker validation is often performed with targeted proteomics methods, such as selected reaction monitoring (SRM)[4] and parallel reaction monitoring (PRM).[5] Recently, biomarker discovery and validation have been increasingly performed

This is an open access article under the CC BY license (http://creativecommons.org/licenses/by/4.0/).

[7]Songjiang Research Institute and Songjiang Hospital, Department of Anatomy and Physiology, College of Basic Medical Science, Shanghai Jiao Tong University School of Medicine, Shanghai 201600, China
[8]Center for Stem Cell Research and Application, Union Hospital, Tongji Medical College, Huazhong University of Science and Technology, Wuhan, Hubei 430074, China
[9]Department of General Surgery, The Second Hospital of Dalian Medical University, Dalian, Liaoning Province 116044, China
[10]Harbin Medical University Cancer Hospital, Harbin, Heilongjiang Province 150081, China
[11]Department of Urology, The Second Hospital of Dalian Medical University, No.467 Zhongshan Road, Dalian, Liaoning Province 116044, China
[12]Department of Clinical Epidemiology, Shengjing Hospital of China Medical University, Shenyang, Liaoning Province 110000, China
[13]The Cancer Hospital of the University of Chinese Academy of Sciences (Zhejiang Cancer Hospital), Hangzhou, Zhejiang 310000, China
[14]Institute of Basic Medicine and Cancer (IBMC), Chinese Academy of Sciences, Hangzhou, Zhejiang 310000, China
[15]College of Mathematics and Computer Science, Zhejiang A & F University, Hangzhou, Zhejiang 311300, China
[16]Lead contact
*Correspondence: c.jimenez@amsterdamumc.nl (C.R.J.), ajun@westlake.edu.cn (J.A.), guotiannan@westlake.edu.cn (T.G.)
https://doi.org/10.1016/j.patter.2023.100792

with targeted analysis of data-independent acquisition (DIA) MS data,[6] an emerging strategy for high-throughput proteomics analyses with a high level of reproducibility.[7] A spectral library containing experimental peptide precursor information is crucial for SRM- and PRM-based protein biomarker validation, as well as DIA-based biomarker discovery.[7] In recent years, spectral libraries have been established for several organisms, such as human,[8,9] mouse,[10] zebrafish,[11] *Arabidopsis thaliana*,[12] and *Escherichia coli*.[13] To support the identification of new protein biomarkers, the comprehensiveness of a spectral library is crucial.

The Human Proteome Project (HPP)[14] launched by the Human Proteome Organization (HUPO) has reported the community-based 10-year achievement of a high-stringency proteome blueprint of 17,874 Protein Evidence 1 (PE1) proteins in 2020, covering 90.4% of the human proteome.[15] A pan-human spectral library (PHL), containing 149,130 peptide precursors and 10,322 proteins, was developed to analyze sequential window acquisition of all theoretical MS (SWATH-MS) data acquired on SCIEX TripleTOF Systems.[8] Another DIA pan-human library (DPHL v.1) for Orbitrap data comprises 289,237 peptide precursors and 10,943 proteins.[9] However, the proteins in these two libraries are proteotypic; protein isoforms are not included. A spectral library with significant coverage of the human proteome and its protein isoforms, with a focus on sequence variations, is thus needed. The use of deep fractionation approaches to build large-scale libraries can significantly increase proteome coverages and allow for the identification of protein isoforms, enabling in-depth proteome profiling. Additionally, previous studies demonstrated that only ∼10%–15% of all the tryptic peptides from a protein sample can be identified when about 50% of the protein identifications are based on a single tryptic peptide due to the intrinsic chemical properties of tryptic peptides.[16–18] Therefore, it will be beneficial to rescue the semi-tryptic peptides.

Here, we present a large DIA spectral library (DPHL v.2), generated from 24 different sample types and available in four variants. DPHL v.2 includes more peptide precursors, peptides, and proteins than DPHL v.1. It also provides higher coverage ratios, particularly for brain-, esophagus-, and ovary-specific or -enriched proteins, as well as FDA-approved drug targets. Two variants of DPHL v.2 generated better identifications of the hallmark gene sets than DPHL v.1. Finally, using a publicly available colorectal cancer (CRC) cohort, DPHL v.2 provided larger numbers of protein and differentially expressed protein identifications than DPHL v.1 and a library-free method.

RESULTS AND DISCUSSION

Data sources for generating DPHL v.2

A total of 1,608 raw MS data files were collected to build our spectral library. Among these, 586 files were newly generated from various samples, including tissue biopsies of prostate cancer (PCa), hepatocellular carcinoma (HCC), triple-negative breast cancer (TNBC), lung adenocarcinoma (LUAD), esophageal carcinoma, thyroid diseases, eyelid tumors, glioblastoma multiforme (GBM), healthy brain tissues, oral squamous cell carcinoma (OSCC), thymic diseases, ovarian cancer (OV), and cervix cancer. Additionally, blood plasma samples from acute myelocytic leukemia (AML), blood diseases, T-lineage acute lymphoblastic leukemia (T-ALL), and healthy plasma exosome were included. Human chronic myelogenous leukemia cell line K562 was also included. Finally, the remaining 1,022 files were derived from the DPHL v.1 study by Zhu et al.[9] The sample types and number of patients contributing to DPHL v.2 are summarized in Figure 1A and Table S1. Both formalin-fixed, paraffin-embedded (FFPE) samples and FF samples were used to build four library variants, as the proteome patterns of these two sample types exhibited a high degree of similarity using peptide samples prepared by the methodology adopted in this study.[19]

Four variants of the pan-human spectral libraries

All 1,608 raw files were centroided and converted into mzXML as previously described.[9] These files were then combined to build our new spectral library. Two different annotation files (i.e., reviewed and isoform-reviewed fasta files) were used to search the mzXML spectra against two digestion modes (i.e., full specific and semi-specific, including both N- and C-terminal semi-tryptic peptides) using MS-Fragger (v.3.0).[20] The reviewed fasta file was obtained from the UniProt database[21] (accessed on July 17, 2020); it included 20,350 reviewed human proteins and was used as the reference. The isoform-reviewed annotation file was also downloaded from UniProt (accessed on August 5, 2020) and comprised 42,347 proteins, including 21,997 human isoforms. Philosopher[22] (v.3.2.9) was used for library searching (using the entire set of 1,608 files) based on the spectra matches with a maximum of two missed cleavages and a global false discovery rate <0.01 for spectra, peptides, and proteins. Of note, the false discovery rates (FDRs) were controlled for large datasets.[23] All other parameters were kept to their default values. By differently combining the two annotation files and the two digestion modes, we generated four library variants: RF

CellPress
OPEN ACCESS

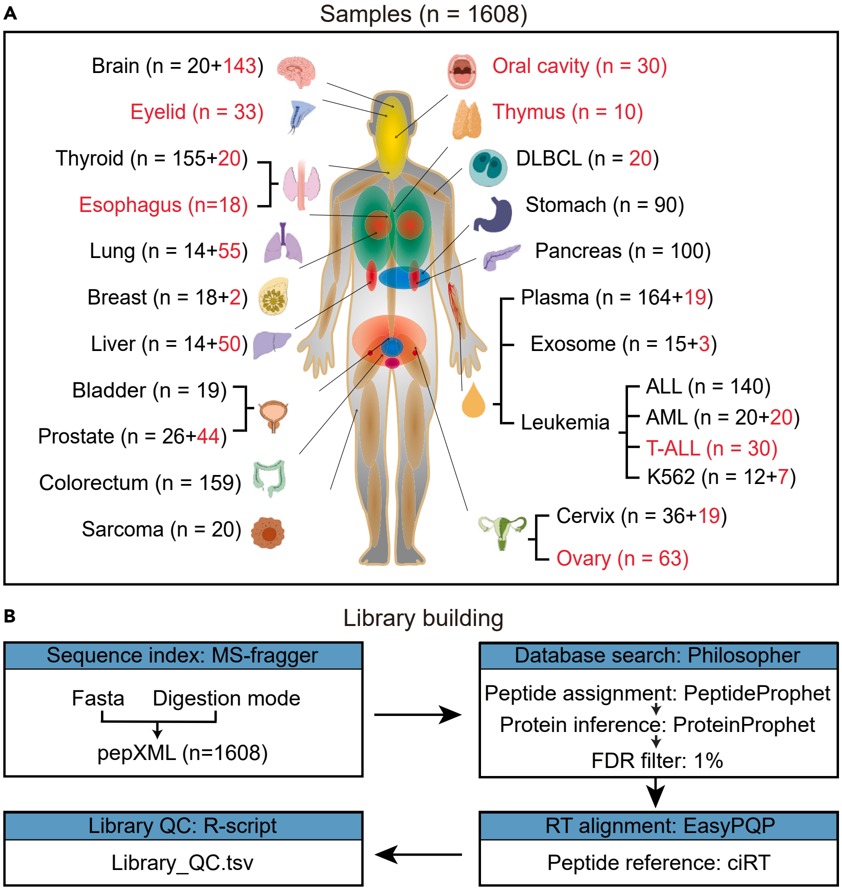

C

Summary of 4 libraries

Fasta	Digestion mode	Proteins	Peptides	Precursors	Name
Reviewed 20,350 proteins	Full-specific	13,465	441,141	601,982	RF
	Semi-specific	13,570	588,984	772,401	RS
Isoform-reviewed 42,347 proteins	Full-specific	14,375	443,150	604,748	IF
	Semi-specific	14,555	624,467	808,672	IS

Figure 1. Sample types and workflow for building DPHL v.2
(A) Number and type of samples included in this study. The ones that were absent in DPHL v.1 are highlighted in red.
(B) Computational pipeline for building DPHL v.2.
(C) Overview of the number of identified proteins, peptides, and precursors using our four library variants.

(reviewed fasta sequence and full-specific digestion mode), RS (reviewed fasta sequence and semi-specific digestion mode), IF (isoform fasta sequence and full-specific digestion mode), and IS (isoform fasta sequence and semi-specific digestion mode).

For the retention time alignment, we chose the conserved high-abundance peptides with common internal retention time (CiRT)[24] from EasyPQP (v.0.1.9, https://github.com/grosenberger/easypqp) in DPHL v.2 rather than the synthetic iRT peptides (SiRT) compared with DPHL v.1. The normalized retention time (RT) correlations (+2/+3 states of each peptide) after these filtering steps are shown in Figure S1, and the standard deviations of the RTs (+2/+3 states of each peptide) are shown in Figures S2A and S2B. To further compare the accuracy of RT fitting, we extracted the RTs of 42,310 CiRT peptides provided by EasyPQP from each pepXML library search file and then calculated the Pearson correlation between the RTs and iRT values in each sample of the four libraries. The average correlation coefficient is greater than 0.94 (Figure S2C), indicating high accuracy of RT fitting. Default parameters were used for all software unless otherwise indicated. The computational pipeline is schematized in Figure 1B.

Characteristics of DPHL v.2

We next evaluated DPHL v.2 using DIALib-QC[25] and found that all four variants of our PHL are of high quality (Figures S3–S6). We also characterized the four libraries in terms of peptide and protein identifications. As shown in Figure 1C, the RF library includes 601,982 peptide precursors, 441,141 peptides, and 13,465 proteins, and the IF library includes 604,748 peptide precursors, 443,150 peptides, and 14,375 proteins. IS, another isoform-based library, comprises 808,672 peptide precursors, 624,467 peptides, and 14,555 proteins. Finally, the RS library contains 772,401 peptide precursors, 588,984 peptides, and 13,570 proteins. We then evaluated the protein identifications of the four libraries for each of the 24 sample types. As shown in Figures S7 and S8, the brain had the highest number of total and unique proteins among all sample types, possibly due to the larger number of brain tissues included (n = 163).

Next, we compared our four libraries with the PHL and DPHL v.1 and found that our four libraries exhibited at least 23% and 30.4% increases in the number of identified proteins compared with DPHL v.1 and the PHL, respectively. Among our four libraries, the isoform-based ones (IS and IF) comprise relatively high numbers of proteins (Figure 2A). Similarly, our four libraries exhibit considerably larger numbers of peptide (Figure 2C) and precursor (Figure 2E) identifications when compared with DPHL v.1 and the PHL. In particular, the semi-specific digestion libraries (IS and RS) have the most significant numbers of peptide and precursor identifications. As shown in Figures 2B, 2D, and 2F, the DPHL v.2 libraries shared 7,262 proteins with DPHL v.1 and the PHL, while 89,328 peptide and 103,704 precursors were shared, respectively. Moreover, 1,144 proteins are exclusively identified by the four variants of DPHL v.2, while 165,041 peptides and 253,673 precursors are introduced by DPHL v.2, respectively. These findings indicate that DPHL v.2 provides higher coverage among precursors, peptides, and proteins than DPHL v.1 and the PHL.

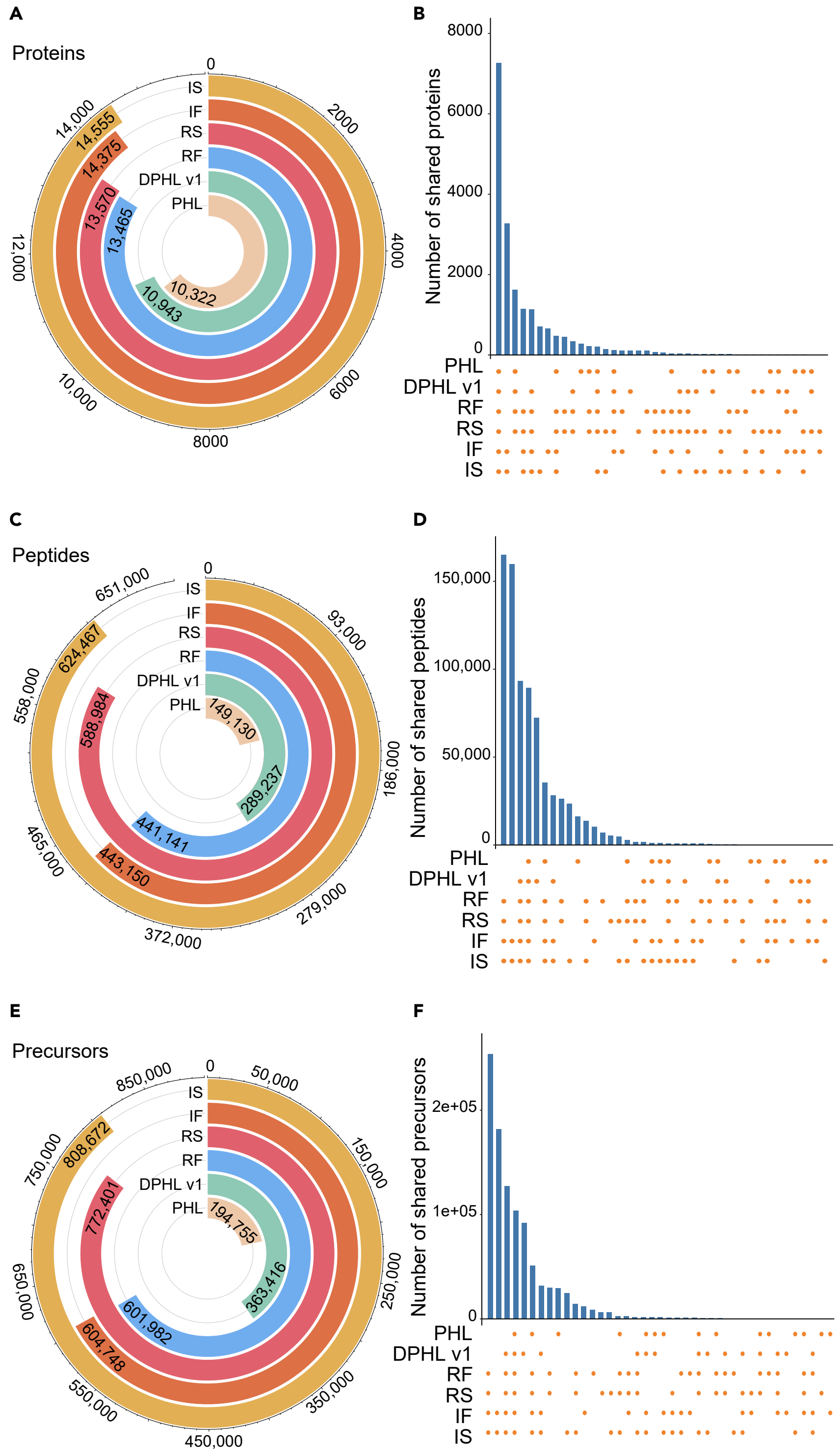

Figure 2. Comparison of the four variants of DPHL v.2 (i.e., RF, RS, IF, and IS) with DPHL v.1 and PHL

(A, C, and E) The circular columns represent the number of proteins (A), peptides (C), or precursor ions (E), with the length of each column proportional to the count.

(B, D, and F) The UpSet plots show the shared and unique protein (B), peptide (D), and precursor identifications (F) of the six libraries.

PHL, pan-human spectral library; DPHL v.1, DIA pan-human library generated by Zhu et al.; RF, reviewed fasta sequence and full-specific digestion mode; RS, reviewed fasta sequence and semi-specific digestion mode; IF, isoform fasta sequence and full-specific digestion mode; IS, isoform fasta sequence and semi-specific digestion mode.

We next compared the numbers of shared proteins and peptides between our four library variants (i.e., between fasta files and digestion models) (Figures 3A and 3B). We found that protein identifications were affected mainly by the fasta file, while peptide identifications were affected by the digestion model. It is not unexpected that the number of protein identifications were mainly affected by the fasta library, as the number of protein sequences determines the protein inference procedure. The semi-specific digestion mode allows identification of large number of semi-tryptic peptides; therefore, it has substantial impact on peptide identification. We also compared our four libraries with DPHL v.1 in terms of the enriched/specific proteins from three tissues (brain, ovary, and esophagus; Figure 3C) obtained from the Human Protein Atlas (https://www.proteinatlas.org/, data available from v21.0.proteinatlas.org). Our results indicated that the coverages of our four libraries are superior to that of DPHL v.1. Similarly, our four libraries provided higher coverage of FDA-approved drug targets than DPHL v.1 (Figure 3C). In addition, the hallmark gene sets from the MSigDB v.7.4 database (http://www.broad.mit.edu/gsea/msigdb/, accessed on November 22, 2021)[26,27] were analyzed using these five libraries. We found that our four libraries cover more than 86% of the genes with well-defined biological states or processes and that both provide better coverage than DPHL v.1 (Figure 3C). 10,552 genes identified in our library have also been characterized by four published datasets (Figure 3D).[23,28–30]

Applicability of DPHL v.2 for DIA-targeted data analysis

To assess the applicability of DPHL v.2, we used our four libraries, DPHL v.1, or a library-free method to analyze a CRC cohort (CRC_1), including 201 CRC cases, 40 benign samples, and 45 biological/technical replicates.[31] The missing values generated by our four libraries or DPHL v.1 were comparable. On the other hand, the library-free method generated fewer missing values (Figure 4A). As shown in Figure 4B, the number of proteins identified with any variant of DPHL v.2 was significantly higher than with DPHL

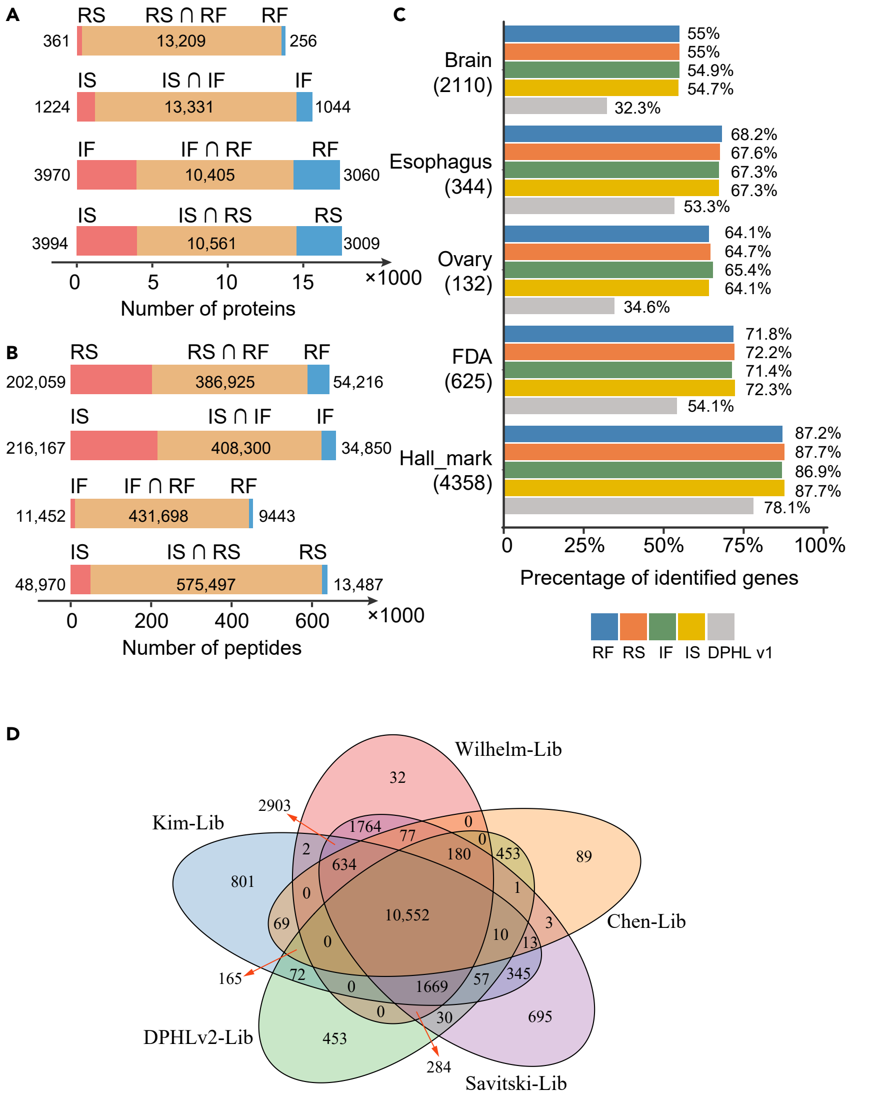

Figure 3. Comparison of the identified proteins in DPHL v.2 with canonical gene sets and other published datasets

(A and B) Comparison of the number of proteins (A) and peptides (B) identified with the same fasta sequence and the same digestion mode.

(C) Percentage of proteins identified among DPHL v.1 and our four libraries using hallmark gene sets, FDA-approved drug targets, and tissue-specific or tissue-enriched/-enhanced proteins from brain, esophagus, and ovary samples.

(D) Comparison of the number of proteins identified with the four other published datasets.

v.1 or the library-free method. A total of 978 dysregulated proteins were identified by all six methods. 1,431 dysregulated proteins were exclusively identified with DPHL v.2 (Figure 4C; Table S2).

In order to demonstrate the applicability of the library, we performed differential expression analyses of the CRC data generated using the six methods described above. Differential expressions were considered significant if their adjusted p values were <0.01 and their $\log_2$ (fold change) absolute values were >1. We obtained 1,997 (RF), 1,984 (RS), 2,024 (IF), 1,992 (IS), 1,783 (DPHL v.1), and 1,737 (library-free) upregulated (adjusted p value < 0.01 and $\log_2$ [fold change] > 1) proteins and 330 (RF), 359 (RS), 346 (IF), 370 (IS), 255 (DPHL v.1), and 230 (library-free) downregulated (adjusted p value < 0.01 and $\log_2$ [fold change] < −1) proteins (Figure 4B; Table S3). Compared with DPHL v.1, the numbers of identified and significantly dysregulated proteins increased by at least 21.7% (RF) and 14.2% (RF). Compared with the analysis using only SwissProt-reviewed protein sequences, 463 and 472 differentially expressed protein isoforms were identified using IF and IS, respectively. Similarly, 94 and 92 proteins were dysregulated in the CRC tissues compared with the benign samples by semi-specific digestion modes. These findings show that DPHL v.2 allows identifying a larger number of differentially expressed proteins or protein isoforms between tumors and benign samples, providing more options for subsequent investigations.

To validate the DIA results, we have included another independent CRC dataset (CRC_2) including 46 samples from patients with CRC.[32] Out of the 2,362 dysregulated proteins identified in CRC using the IF library, 2,013 proteins were also found in the CRC_2 dataset, comprising 374 protein isoforms. Among these isoforms, 68 exhibited differential expressions between tumors and healthy samples, including the DNASE2 isoform (O00115-2) and the SYNM isoform (O15061-2). The DNASE2 isoform exhibited significant upregulation in the patients with CRC, whereas the SYNM isoform was upregulated in healthy samples, consistent with our observations (Figures S9A and S9B). These findings indicate that the DPHL v.2 is a valuable resource for DIA-based discovery of potential biomarkers of CRC.

We next built an XGBoost machine learning model in the RF set based on the dysregulated proteins that were shared across the four library variants. The CRC_1 dataset was randomly divided into a training set (n = 200) and a test set (n = 41). In addition, 40 samples from the training set were randomly selected as an internal validation set to optimize the model's parameters. This process of selecting an internal validation set has been iterated ten times. We also stepwise modified multiple key parameters including "eta," "subsample," and "gamma," as well as the number of protein features. More details are provided in the experimental procedures section. A total of 9,600 models were generated, of which 960 showed an accuracy (ACC) and area under the curve (AUC) of 1 in the internal validation set. These models were then tested in the test set and yielded ACC values greater than 0.9. Among the 960 models, 542 had AUC values higher than 0.9. We then selected one with the following specific parameters: eta = 0.25, subsample = 1, and gamma = 0.2, which included 14 features, such as SNCG, S100B, CEACAM6, OGN,

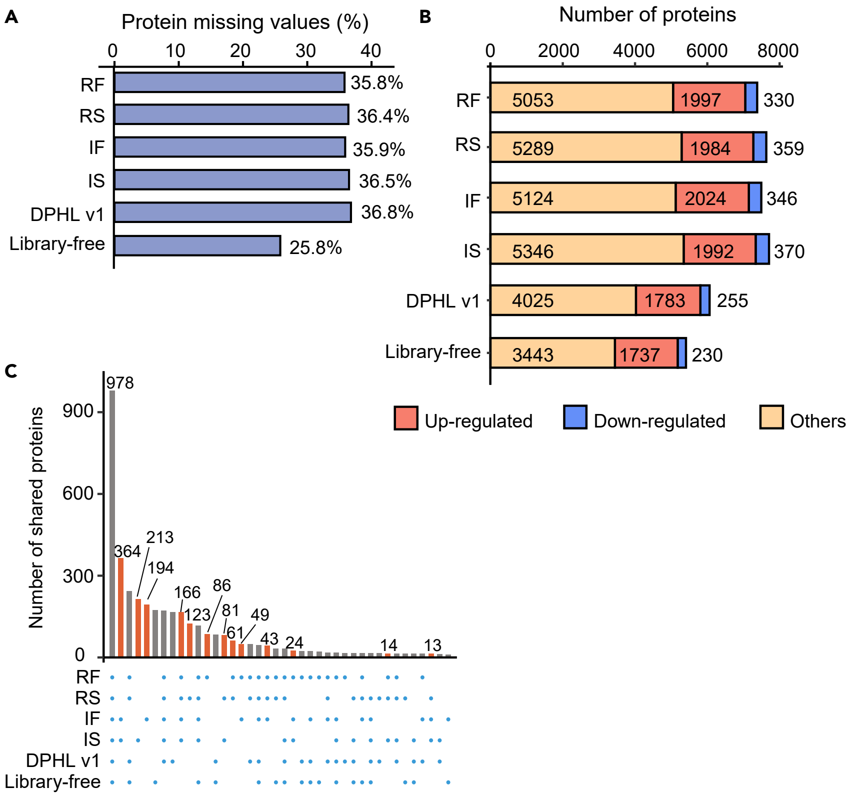

Figure 4. DIA analysis of CRC and benign samples

(A) Number of protein missing values obtained using the five libraries and library-free method.

(B) Number of differentially expressed proteins between CRC and benign samples obtained using the five libraries and library-free method. Proteins with adjusted p value <0.01 and |FC| >4 were selected as significantly differentially expressed. FC, fold change.

(C) Dysregulated protein identification overlaps across the six libraries. The orange bars indicate the proteins identified only by DPHL v.2 libraries.

NNMT, SH3PXD2B, IGFBP7, SERPINH1, CDYL2, TIMP1, APOOL, SMARCD2, IGHG4, and F2. The importance values of these 14 features are shown in Figure 5A. The AUCs of the training set and the test set achieved 1 and 0.943, respectively (Figures 5B and 5C), while the ACCs achieved 1 and 0.927, respectively (Figures 5E and 5F). The model was further validated in four datasets (RS, IF, IS, and CRC_2), achieving AUC values of 0.971, 0.99, 0.981, and 0.94, respectively (Figures S9C–S9E and 5D), while the ACC values were 0.979, 0.979, 0.975, and 0.867, respectively (Figure S9F–S9H and 5G). These results indicate the effectiveness and robustness of this model. Among the proteins prioritized in this model, multiple proteins have been reported to be closely related to CRC. They include SNCG,[33,34] S100B,[35] CEACAM6,[36,37] OGN,[38] NNMT,[39,40] IGFBP7,[41] SERPINH1,[42] CDYL2,[43] TIMP1,[44] and F2.[24] Four proteins, namely SH3PXD2B, APOOL, SMARCD2, and IGHG4, previously not associated with CRC are also identified. Furthermore, following the method described above, we developed a machine learning model based on all differentially expressed proteins from the RF set. The model and its performance were similar to those of overlapped differentially expressed proteins based on four libraries (Figures S10A–S10C).

Analysis of protein isoforms and semi-tryptic peptides

We next checked whether this resource could be used to analyze specific protein isoforms. Among the dysregulated proteins from IF, we identified SPTBN1 (SPTBN1-long) and one of its isoforms (SPTBN1-short).[45] As reported in literature, SPTBN1 is significantly dysregulated and plays an essential role in liver cancer,[46] CRC, and breast cancer, among others.[47,48] We further examined the protein sequences of the two isoforms, namely SPTBN1-long and SPTBN-short as shown in Figure 6. In addition to the common parts of the two sequences, our library also identified the peptide (TSSISGPLSPAYTGQVPYNYNQLEGR) specific in SPTBN1-short (Figure 6A). Skyline software (Skyline-daily version, downloaded on August 3, 2020) was used to show that the peak spectrum of this peptide and a common peptide form these two proteins within the DIA raw file (Figures 6B and 6C).

Regarding those that were only characterized through semi-specific peptides in our semi-specific libraries (IS and RS), including VWF, LMO7, ALDH2, NPEPL1, NUAK1, and TPT1, many of them have important biologic implications. ADAM22 is a new therapeutic option for treating metastatic brain disease and may be appropriate for the treatment of breast cancer.[49,50] By analyzing mRNA expression profiles, Xin et al. found that *ASPM* is highly expressed in GBM and that patients with high *ASPM* expression have poor prognoses.[51] LRP6 inhibits cell proliferation and delays tumor growth *in vivo*, especially in colon, liver, breast, and pancreatic cancers.[52,53] CHD9 was reported as a potential biomarker for clear cell renal cell carcinoma.[54] In addition, FAIM2 promotes non-small cell lung cancer growth and bone metastasis formation by regulating the epithelial-mesenchymal transformation process and the Wnt/β-catenin signaling pathway.[55] In our analysis, all these proteins showed significant differences between tumor and non-tumor samples, indicating that DPHL v.2 can assist with the discovery of new potential protein biomarkers.

Conclusion

We present DPHL v.2, which contains four comprehensive spectral libraries (RF, RS, IF, and IS) derived from 1,608 DDA-MS raw files, including 24 sample types. Covering over 440,000 peptides and more than 14,000 proteins, DPHL v.2 can confidently detect and quantify more than 66.1% of the reviewed human proteins annotated by UniProtKB/SwissProt. Our results suggest that DPHL v.2 could support protein biomarker identification, especially for protein isoforms and semi-tryptic peptides. DPHL v.2 outperforms previous DIA libraries in the following aspects. Firstly, five

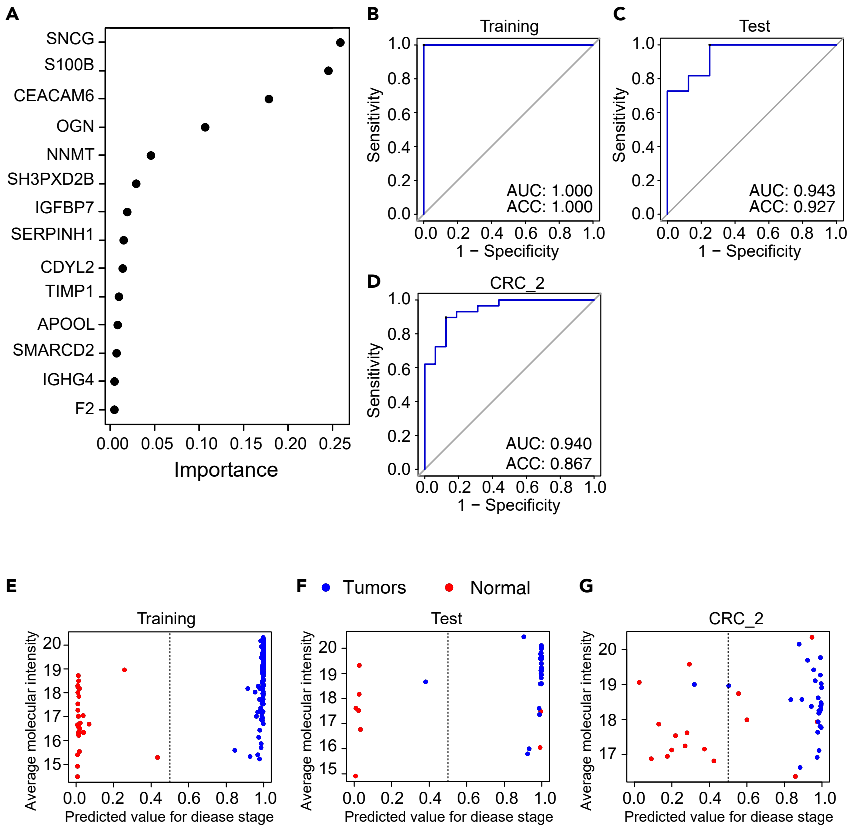

Figure 5. Machine learning to identify potential CRC biomarkers

(A) Prioritization of 14 important variables.

(B–D) Receiver operating characteristic (ROC) plots for the training set, the test set, and the CRC_2 dataset.

(E–G) Performance of the model in the training set, the test set, and the CRC_2 dataset.

additional tissue types (oral cavity, thymus, esophagus, eyelid, and ovary) and one blood plasma sample from T-ALL were included. Secondly, protein isoforms and semi-trypsin digestion were used for library searching. In addition, these libraries are compatible with various commonly used DIA tools, with or without format transformation, such as OpenSWATH,[56] DIA-NN,[57] Skyline,[58] and Spectronaut.[59]

These libraries should be used in different scenarios. To analyze protein isoforms, one should use the two isoform-based library variants. Semi-annotated libraries (IS and RS) may be more appropriate for primary tissue samples, especially those that are susceptible to degradation. For comprehensive searches aiming for maximum protein identifications, we suggest using the isoform-semi library (IF), as it contains the highest number of protein entries. On the other hand, for well-established cell line samples, the reviewed-full library (RF) is recommended because of its reduced search space.

EXPERIMENTAL PROCEDURES

Resource availability

Lead contact

Further information should be directed to and will be fulfilled by the lead contact, Tiannan Guo (guotiannan@westlake.edu.cn).

Materials availability

This study did not generate new unique materials.

Data and code availability

All newly added raw DDA-MS data (in mzXML format), four spectral libraries (in tsv files), and two types of fasta files (reviewed fasta and isoform fasta) have been deposited to the ProteomeXchange Consortium (http://proteomecentral.proteomexchange.org) via the iProX partner repository iProX[60] (iProX: IPX0005714000) with the dataset identifier PXD039313. The sample type and acquisition method for each MS raw file are detailed in Table S1. All original code has been deposited at Zenodo under https://doi.org/10.5281/zenodo.7998229 and is publicly available as of the date of publication.

Materials

All chemicals used in this study were purchased from Sigma. All MS-grade reagents were acquired from Thermo Fisher Scientific (Waltham, MA, USA).

Clinical samples

FFPE, fresh or fresh frozen tissue biopsies from GBM, healthy human brain, eyelid tumor, thyroid disease, sarcoma, OSCC, thymus, LUAD, TNBC, HCC, gastric cancer, diffuse large B cell lymphoma (DLBCL), pancreatic ductal adenocarcinoma, bladder cancer, PCa, and OV were collected in this study. Human plasma samples, including ALL, AML, T-ALL, healthy plasma exosome, and blood disease, were also analyzed, as well as K562 cells. Six of these tissues were new additions compared with DPHL v.1. Eyelid samples were obtained from the Second Affiliated Hospital of Zhejiang University School of Medicine, China. The ovary cohort was obtained from The Cancer Hospital of the University of Chinese Academy of Sciences. The OSCC, esophagus, T-ALL, and thymus cancer samples were collected at Amsterdam UMC/VU Medical Center, Amsterdam, and Erasmus University Medical Center. Sample details are provided in Table S1.

To compare our libraries with DPHL v.1 and the library-free method, we used the DIA data of a CRC cohort generated by Ge et al.,[31] which consists of 201 cancer samples, 40 para-cancer tissues, and 45 biological and technical replicates from 40 patients with CRC and four healthy controls. The CRC cohort was not included to build the four libraries but only to evaluate the applicability of DPHL v.2.

QC control of the library

In this study, precursor refers to the ionized form of a peptide that has an associated charge. A peptide is defined as a unique sequence of amino acids with potential modifications, such as post-translational modifications or chemical modifications. Protein groups are a set of proteins with shared peptide sequences. Here, proteins indicate the top ranking protein accession ID from a protein group determined by FragPipe. Quality controls (QCs) were then performed using an R script with the criteria next described to remove data of low quality. First, only precursors with multiple fragments ($\geq$2) and a normalized RT range from -60 to 200 were retained. Here, the RT value might be negative because it has been normalized to a set of CiRT peptides. Second, fragments with a library intensity <10 or a precursor charge of +1 were removed. Finally, peptides with only one precursor were retained. However, when a peptide has more than two precursors, the average normalized RTs of all precursors and their differences with respect to their mean RT were calculated. Next, peptides with an absolute normalized RT difference >5 were excluded. When all the absolute values were >5, the median normalized RT of all the precursors and their difference from the median normalized RT were calculated: only the peptides with an absolute normalized RT difference <5 were then selected.

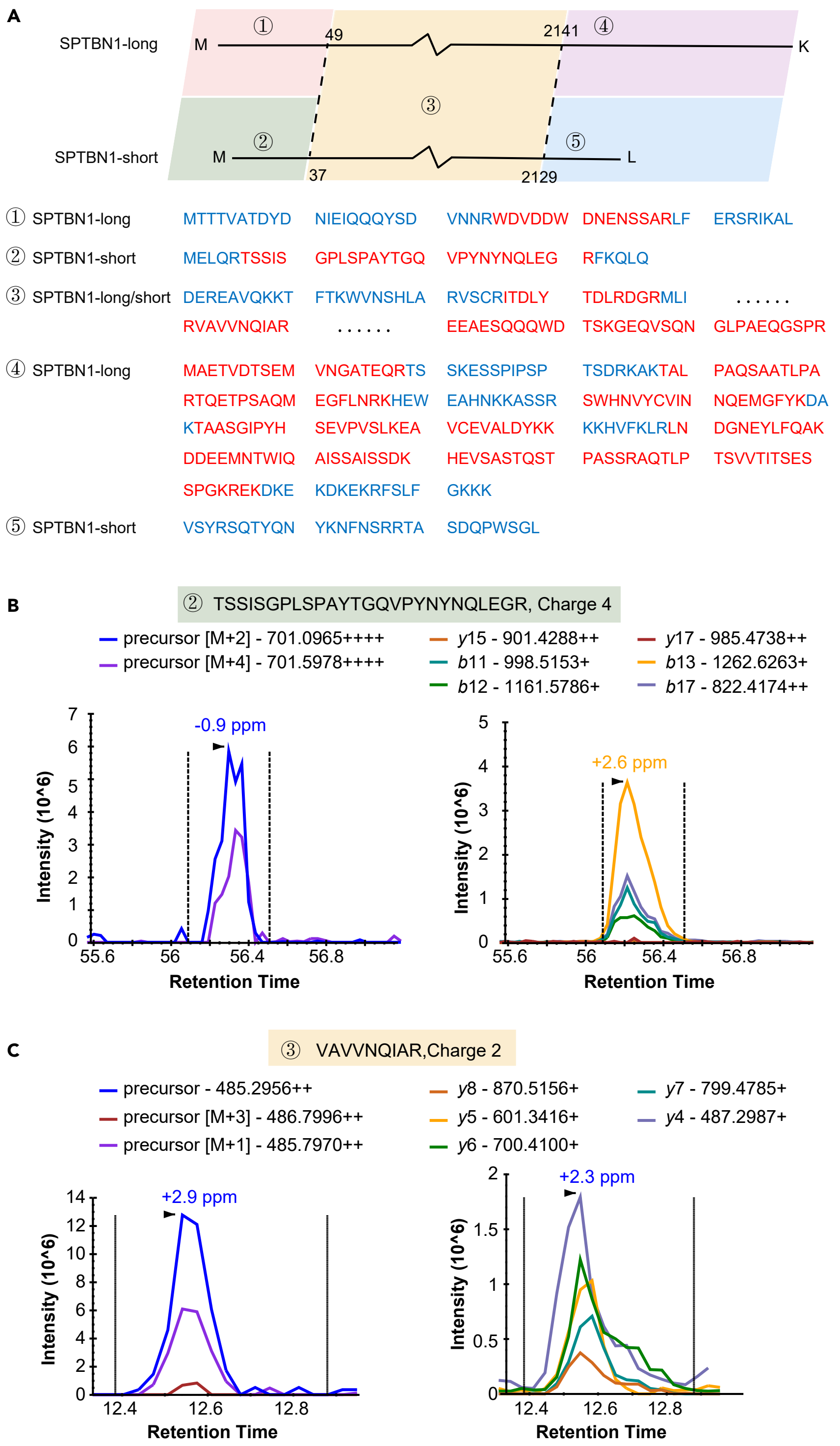

Figure 6. SPTBN1 protein identification in our DIA search results

(A) Sequences of SPTBN1 and its isoform. Blue: sequences that were not identified; red: identified sequences.

(B) The peak spectrum of peptide SSISGPLSPAYTGQVPYNYNQLEGR in our DIA raw file (obtained using Skyline).

(C) The peak spectrum of peptide VAVVNQIAR in our DIA raw file (obtained using Skyline).

MS data acquisition

Among the newly added 586 DDA raw data files, 108 were derived from Dutch cohorts generated at the Jimenez lab and 404 from Chinese cohorts generated at the Guo lab. The pipeline for generating these DDA files coincided with that used for DPHL v.1. The DDA raw files were centroided and converted into mzXML using ProteoWizard[61] (v.3.0.11579). We used FragPipe v.3.0 for spectral data analysis with carbamidomethylation as a fixed modification at cysteine residues and an oxidation set as a variable modification at methionine residues. The mass ACC was set as 20 ppm. Peptides with 7–50 amino acids were included in the analysis, with a maximal missed cleavage of 2.

DIA data analysis

The DIA raw files were submitted to DIA-NN (1.7.15), a tool for DIA or SWATH proteomics data analysis.[57] Our four libraries were used as a reference, and no other fasta sequences were added. The library inference was set to "off." All

other parameters were kept to their default values. The tools we used for the DIA data analysis, as described above, are publicly available.[57]

Machine learning

The XGBoost analysis was performed with the R package "xgboost" (v.1.6.0.1). 1,426 proteins were firstly selected as input features to build the XGBoost model. The eta was set from 0.2 to 0.3, with a step size of 0.05. The subsample was set from 0.8 to 1, with a step size of 0.05. The gamma was set from 0.05 to 0.2, with a step size of 0.05. The number of protein features were set from 5 to 20, with a step size of 1. The final performance was evaluated by mean ACC and mean AUC.

Ethical statement

Ethics approvals for this study were obtained from the ethics committee or institutional review board of each participating institution.

SUPPLEMENTAL INFORMATION

Supplemental information can be found online at https://doi.org/10.1016/j.patter.2023.100792.

ACKNOWLEDGMENTS

This work is supported by a grant from the National Key R&D Program of China (2022YF0608403).

AUTHOR CONTRIBUTIONS

T.G. conceived the project. Z.X. and T.Z. built all the libraries. Z.X. processed and analyzed data. T.G., Z.X., T.Z., J.A., and F.Z. wrote the manuscript. Y.L. collected the brain samples. J.Y. provided the eyelid tumor samples. T.L. offered the lung cancer samples. J.Z. and C.L. collected the liver cancer samples. Y.H. offered the PCa samples. Q.W. provided the cervix cancer samples. J.Z. and Z.Z. collected the OV samples. Others prepared peptides for the study. T.G. supervised the work. All authors reviewed and approved the manuscript.

DECLARATION OF INTERESTS

T.G. is shareholder of Westlake Omics, Inc. N.X., X.Y., and W.L. were employees of Westlake Omics, Inc., when they participated in this project.

DECLARATION OF GENERATIVE AI AND AI-ASSISTED TECHNOLOGIES IN THE WRITING PROCESS

During the preparation of this work, the author(s) used ChatGPT, an AI language model, for language refinement. The generated text was carefully reviewed and edited by the authors to ensure its accuracy and appropriateness. The authors take full responsibility for the final content of the publication and acknowledge the valuable assistance provided by AI technology in the language polishing process.

Received: January 6, 2023
Revised: April 29, 2023
Accepted: June 12, 2023
Published: July 5, 2023

REFERENCES

1. Zhu, Y., Aebersold, R., Mann, M., and Guo, T. (2021). SnapShot: Clinical proteomics. Cell *184*, 4840–4840.e1. https://doi.org/10.1016/j.cell.2021.08.015.
2. Xiao, Q., Zhang, F., Xu, L., Yue, L., Kon, O.L., Zhu, Y., and Guo, T. (2021). High-throughput proteomics and AI for cancer biomarker discovery. Adv. Drug Deliv. Rev. *176*, 113844. https://doi.org/10.1016/j.addr.2021.113844.
3. Aebersold, R., and Mann, M. (2016). Mass-spectrometric exploration of proteome structure and function. Nature *537*, 347–355. https://doi.org/10.1038/nature19949.
4. Lange, V., Picotti, P., Domon, B., and Aebersold, R. (2008). Selected reaction monitoring for quantitative proteomics: a tutorial. Mol. Syst. Biol. *4*, 222. https://doi.org/10.1038/msb.2008.61.
5. Peterson, A.C., Russell, J.D., Bailey, D.J., Westphall, M.S., and Coon, J.J. (2012). Parallel reaction monitoring for high resolution and high mass accuracy quantitative, targeted proteomics. Mol. Cell. Proteomics *11*, 1475–1488. https://doi.org/10.1074/mcp.O112.020131.
6. Gillet, L.C., Navarro, P., Tate, S., Röst, H., Selevsek, N., Reiter, L., Bonner, R., and Aebersold, R. (2012). Targeted data extraction of the MS/MS spectra generated by data-independent acquisition: a new concept for consistent and accurate proteome analysis. Mol. Cell. Proteomics *11*, O111.016717. https://doi.org/10.1074/mcp.O111.016717.
7. Zhang, F., Ge, W., Ruan, G., Cai, X., and Guo, T. (2020). Data-Independent Acquisition Mass Spectrometry-Based Proteomics and Software Tools: A Glimpse in 2020. Proteomics *20*, e1900276. https://doi.org/10.1002/pmic.201900276.
8. Rosenberger, G., Koh, C.C., Guo, T., Röst, H.L., Kouvonen, P., Collins, B.C., Heusel, M., Liu, Y., Caron, E., Vichalkovski, A., et al. (2014). A repository of assays to quantify 10,000 human proteins by SWATH-MS. Sci. Data *1*, 140031. https://doi.org/10.1038/sdata.2014.31.
9. Zhu, T., Zhu, Y., Xuan, Y., Gao, H., Cai, X., Piersma, S.R., Pham, T.V., Schelfhorst, T., Haas, R.R.G.D., Bijnsdorp, I.V., et al. (2020). DPHL: A DIA Pan-human Protein Mass Spectrometry Library for Robust Biomarker Discovery. Dev. Reprod. Biol. *18*, 104–119. https://doi.org/10.1016/j.gpb.2019.11.008.
10. Lu, T., Qian, L., Xie, Y., Zhang, Q., Liu, W., Ge, W., Zhu, Y., Ma, L., Zhang, C., and Guo, T. (2022). Tissue-Characteristic Expression of Mouse Proteome. Mol. Cell. Proteomics *21*, 100408. https://doi.org/10.1016/j.mcpro.2022.100408.
11. Blattmann, P., Stutz, V., Lizzo, G., Richard, J., Gut, P., and Aebersold, R. (2019). Generation of a zebrafish SWATH-MS spectral library to quantify 10,000 proteins. Sci. Data *6*, 190011. https://doi.org/10.1038/sdata.2019.11.
12. Zhang, H., Liu, P., Guo, T., Zhao, H., Bensaddek, D., Aebersold, R., and Xiong, L. (2019). Arabidopsis proteome and the mass spectral assay library. Sci. Data *6*, 278. https://doi.org/10.1038/s41597-019-0294-0.
13. Midha, M.K., Kusebauch, U., Shteynberg, D., Kapil, C., Bader, S.L., Reddy, P.J., Campbell, D.S., Baliga, N.S., and Moritz, R.L. (2020). A comprehensive spectral assay library to quantify the Escherichia coli proteome by DIA/SWATH-MS. Sci. Data *7*, 389. https://doi.org/10.1038/s41597-020-00724-7.
14. Omenn, G.S., Lane, L., Overall, C.M., Pineau, C., Packer, N.H., Cristea, I.M., Lindskog, C., Weintraub, S.T., Orchard, S., Roehrl, M.H.A., et al. (2023). The 2022 Report on the Human Proteome from the HUPO Human Proteome Project. J. Proteome Res. *22*, 1024–1042. https://doi.org/10.1021/acs.jproteome.2c00498.
15. Adhikari, S., Nice, E.C., Deutsch, E.W., Lane, L., Omenn, G.S., Pennington, S.R., Paik, Y.K., Overall, C.M., Corrales, F.J., Cristea, I.M., et al. (2020). A high-stringency blueprint of the human proteome. Nat. Commun. *11*, 5301. https://doi.org/10.1038/s41467-020-19045-9.
16. Mallick, P., Schirle, M., Chen, S.S., Flory, M.R., Lee, H., Martin, D., Ranish, J., Raught, B., Schmitt, R., Werner, T., et al. (2007). Computational prediction of proteotypic peptides for quantitative proteomics. Nat. Biotechnol. *25*, 125–131. https://doi.org/10.1038/nbt1275.
17. Tang, H., Arnold, R.J., Alves, P., Xun, Z., Clemmer, D.E., Novotny, M.V., Reilly, J.P., and Radivojac, P. (2006). A computational approach toward label-free protein quantification using predicted peptide detectability. Bioinformatics *22*, e481–e488. https://doi.org/10.1093/bioinformatics/btl237.
18. States, D.J., Omenn, G.S., Blackwell, T.W., Fermin, D., Eng, J., Speicher, D.W., and Hanash, S.M. (2006). Challenges in deriving high-confidence protein identifications from data gathered by a HUPO plasma proteome

collaborative study. Nat. Biotechnol. *24*, 333–338. https://doi.org/10.1038/nbt1183.

19. Zhu, Y., Weiss, T., Zhang, Q., Sun, R., Wang, B., Yi, X., Wu, Z., Gao, H., Cai, X., Ruan, G., et al. (2019). High-throughput proteomic analysis of FFPE tissue samples facilitates tumor stratification. Mol. Oncol. *13*, 2305–2328. https://doi.org/10.1002/1878-0261.12570.
20. Kong, A.T., Leprevost, F.V., Avtonomov, D.M., Mellacheruvu, D., and Nesvizhskii, A.I. (2017). MSFragger: ultrafast and comprehensive peptide identification in mass spectrometry-based proteomics. Nat. Methods *14*, 513–520. https://doi.org/10.1038/nmeth.4256.
21. Magrane, M., and UniProt, C. (2011). UniProt Knowledgebase: a hub of integrated protein data, p. bar009. Database. https://doi.org/10.1093/database/bar009.
22. da Veiga Leprevost, F., Haynes, S.E., Avtonomov, D.M., Chang, H.Y., Shanmugam, A.K., Mellacheruvu, D., Kong, A.T., and Nesvizhskii, A.I. (2020). Philosopher: a versatile toolkit for shotgun proteomics data analysis. Nat. Methods *17*, 869–870. https://doi.org/10.1038/s41592-020-0912-y.
23. Savitski, M.M., Wilhelm, M., Hahne, H., Kuster, B., and Bantscheff, M. (2015). A Scalable Approach for Protein False Discovery Rate Estimation in Large Proteomic Data Sets. Mol. Cell. Proteomics *14*, 2394–2404. https://doi.org/10.1074/mcp.M114.046995.
24. Zhang, T., Guo, J., Gu, J., Wang, Z., Wang, G., Li, H., and Wang, J. (2019). Identifying the key genes and microRNAs in colorectal cancer liver metastasis by bioinformatics analysis and in vitro experiments. Oncol. Rep. *41*, 279–291. https://doi.org/10.3892/or.2018.6840.
25. Midha, M.K., Campbell, D.S., Kapil, C., Kusebauch, U., Hoopmann, M.R., Bader, S.L., and Moritz, R.L. (2020). DIALib-QC an assessment tool for spectral libraries in data-independent acquisition proteomics. Nat. Commun. *11*, 5251. https://doi.org/10.1038/s41467-020-18901-y.
26. Subramanian, A., Tamayo, P., Mootha, V.K., Mukherjee, S., Ebert, B.L., Gillette, M.A., Paulovich, A., Pomeroy, S.L., Golub, T.R., Lander, E.S., et al. (2005). Gene set enrichment analysis a knowledge-based approach for interpreting genome-wide expression profiles. Proc. Natl. Acad. Sci. USA *102*, 15545–15550. https://doi.org/10.1073/pnas.0506580102.
27. Liberzon, A., Birger, C., Thorvaldsdóttir, H., Ghandi, M., Mesirov, J.P., and Tamayo, P. (2015). The Molecular Signatures Database (MSigDB) hallmark gene set collection. Cell Syst. *1*, 417–425. https://doi.org/10.1016/j.cels.2015.12.004.
28. Wilhelm, M., Schlegl, J., Hahne, H., Gholami, A.M., Lieberenz, M., Savitski, M.M., Ziegler, E., Butzmann, L., Gessulat, S., Marx, H., et al. (2014). Mass-spectrometry-based draft of the human proteome. Nature *509*, 582–587. https://doi.org/10.1038/nature13319.
29. Kim, M.S., Pinto, S.M., Getnet, D., Nirujogi, R.S., Manda, S.S., Chaerkady, R., Madugundu, A.K., Kelkar, D.S., Isserlin, R., Jain, S., et al. (2014). A draft map of the human proteome. Nature *509*, 575–581. https://doi.org/10.1038/nature13302.
30. Chen, F., Chandrashekar, D.S., Varambally, S., and Creighton, C.J. (2019). Pan-cancer molecular subtypes revealed by mass-spectrometry-based proteomic characterization of more than 500 human cancers. Nat. Commun. *10*, 5679. https://doi.org/10.1038/s41467-019-13528-0.
31. Ge, W., Liang, X., Zhang, F., Hu, Y., Xu, L., Xiang, N., Sun, R., Liu, W., Xue, Z., Yi, X., et al. (2021). Computational Optimization of Spectral Library Size Improves DIA-MS Proteome Coverage and Applications to 15 Tumors. J. Proteome Res. *20*, 5392–5401. https://doi.org/10.1021/acs.jproteome.1c00640.
32. Shao, Y., Xu, K., Zheng, X., Zhou, B., Zhang, X., Wang, L., Sun, Y., Li, D., Chen, T., Wang, J., et al. (2022). Proteomics profiling of colorectal cancer progression identifies PLOD2 as a potential therapeutic target. Cancer Commun. *42*, 164–169. https://doi.org/10.1002/cac2.12240.
33. Hu, H., Sun, L., Guo, C., Liu, Q., Zhou, Z., Peng, L., Pan, J., Yu, L., Lou, J., Yang, Z., et al. (2009). Tumor cell-microenvironment interaction models coupled with clinical validation reveal CCL2 and SNCG as two predictors of colorectal cancer hepatic metastasis. Clin. Cancer Res. *15*, 5485–5493. https://doi.org/10.1158/1078-0432.CCR-08-2491.
34. Liu C, D.B., Lu, A., Qu, L., Xing, X., Meng, L., Wu, J., Eric Shi, Y., and Shou, C. (2010). Synuclein gamma predicts poor clinical outcome in colon cancer with normal levels of carcinoembryonic antigen. BMC Cancer *359*, 1471–2407.
35. Huang, M.Y., Wang, H.M., Tok, T.S., Chang, H.J., Chang, M.S., Cheng, T.L., Wang, J.Y., and Lin, S.R. (2012). EVI2B, ATP2A2, S100B, TM4SF3, and OLFM4 as potential prognostic markers for postoperative Taiwanese colorectal cancer patients. DNA Cell Biol. *31*, 625–635. https://doi.org/10.1089/dna.2011.1365.
36. Ferlizza, E., Solmi, R., Miglio, R., Nardi, E., Mattei, G., Sgarzi, M., and Lauriola, M. (2020). Colorectal cancer screening: Assessment of CEACAM6, LGALS4, TSPAN8 and COL1A2 as blood markers in faecal immunochemical test negative subjects. J. Adv. Res. *24*, 99–107. https://doi.org/10.1016/j.jare.2020.03.001.
37. Rodia, M.T., Solmi, R., Pasini, F., Nardi, E., Mattei, G., Ugolini, G., Ricciardiello, L., Strippoli, P., Miglio, R., and Lauriola, M. (2018). LGALS4, CEACAM6, TSPAN8, and COL1A2: Blood Markers for Colorectal Cancer-Validation in a Cohort of Subjects With Positive Fecal Immunochemical Test Result. Clin. Colorectal Cancer *17*, e217–e228. https://doi.org/10.1016/j.clcc.2017.12.002.
38. Hu, X., Li, Y.Q., Li, Q.G., Ma, Y.L., Peng, J.J., and Cai, S.J. (2018). Osteoglycin (OGN) reverses epithelial to mesenchymal transition and invasiveness in colorectal cancer via EGFR/Akt pathway. J. Exp. Clin. Cancer Res. *37*, 41. https://doi.org/10.1186/s13046-018-0718-2.
39. Li, G., Fang, S., Shao, X., Li, Y., Tong, Q., Kong, B., Chen, L., Wang, Y., Yang, J., Yu, H., et al. (2021). Curcumin Reverses NNMT-Induced 5-Fluorouracil Resistance via Increasing ROS and Cell Cycle Arrest in Colorectal Cancer Cells. Biomolecules *11*, 1295. https://doi.org/10.3390/biom11091295.
40. Song, M., Li, Y., Miao, M., Zhang, F., Yuan, H., Cao, F., Chang, W., Shi, H., and Song, C. (2020). High stromal nicotinamide N-methyltransferase (NNMT) indicates poor prognosis in colorectal cancer. Cancer Med. *9*, 2030–2038. https://doi.org/10.1002/cam4.2890.
41. Qiu, B., Chu, L.Y., Li, X.X., Peng, Y.H., Xu, Y.W., Xie, J.J., and Chen, X.Y. (2020). Diagnostic Value of Serum Insulin-Like Growth Factor Binding Protein 7 (IGFBP7) in Colorectal Cancer. OncoTargets Ther. *13*, 12131–12139. https://doi.org/10.2147/OTT.S266478.
42. Zhang, Y., Li, C.Y., Ge, W., and Xiao, Y. (2021). Exploration of the Key Proteins in the Normal-Adenoma-Carcinoma Sequence of Colorectal Cancer Evolution Using In-Depth Quantitative Proteomics. J. Oncol. *2021*, 5570058. https://doi.org/10.1155/2021/5570058.
43. Kim, S.T., Sohn, I., Jang, J., Jang, J., Kim, S.H., Jung, I.H., Park, J.O., Park, Y.S., Talasaz, A., Lee, J., and Kim, H.C. (2014). Transcriptome analysis of CD133-positive stem cells and prognostic value of survivin in colorectal cancer. Cancer Genomics Proteomics *11*, 259–266.
44. Song, G., Xu, S., Zhang, H., Wang, Y., Xiao, C., Jiang, T., Wu, L., Zhang, T., Sun, X., Zhong, L., et al. (2016). TIMP1 is a prognostic marker for the progression and metastasis of colon cancer through FAK-PI3K/AKT and MAPK pathway. J. Exp. Clin. Cancer Res. *35*, 148. https://doi.org/10.1186/s13046-016-0427-7.
45. Hayes, N., Scott, C., Heerkens, E., Ohanian, V., Maggs, A., Pinder, J., Kordeli, E., and Baines, A. (2000). Identification of a novel C-terminal variant of βII spectrin two isoforms of βII spectrin have distinct intracellular locations and activities. J. Cell Sci. *113*, 2023–2034.
46. Rao, S., Yang, X., Ohshiro, K., Zaidi, S., Wang, Z., Shetty, K., Xiang, X., Hassan, M.I., Mohammad, T., Latham, P.S., et al. (2021). β2-spectrin (SPTBN1) as a therapeutic target for diet-induced liver disease and preventing cancer development. Sci. Transl. Med. *13*, eabk2267.
47. Yang, P., Yang, Y., Sun, P., Tian, Y., Gao, F., Wang, C., Zong, T., Li, M., Zhang, Y., Yu, T., and Jiang, Z. (2021). betaII spectrin (SPTBN1): biological function and clinical potential in cancer and other diseases. Int. J. Biol. Sci. *17*, 32–49. https://doi.org/10.7150/ijbs.52375.
48. Yao, Z.X., Jogunoori, W., Choufani, S., Rashid, A., Blake, T., Yao, W., Kreishman, P., Amin, R., Sidawy, A.A., Evans, S.R.T., et al. (2010). Epigenetic silencing of beta-spectrin, a TGF-beta signaling/scaffolding

CellPress
OPEN ACCESS

protein in a human cancer stem cell disorder: Beckwith-Wiedemann syndrome. J. Biol. Chem. *285*, 36112–36120. https://doi.org/10.1074/jbc.M110.162347.

49. Charmsaz, S., Doherty, B., Cocchiglia, S., Varešlija, D., Marino, A., Cosgrove, N., Marques, R., Priedigkeit, N., Purcell, S., Bane, F., et al. (2020). ADAM22/LGI1 complex as a new actionable target for breast cancer brain metastasis. BMC Med. *18*, 349. https://doi.org/10.1186/s12916-020-01806-4.

50. Li, J., Lu, M., Jin, J., Lu, X., Xu, T., and Jin, S. (2018). miR-449a Suppresses Tamoxifen Resistance in Human Breast Cancer Cells by Targeting ADAM22. Cell. Physiol. Biochem. *50*, 136–149. https://doi.org/10.1159/000493964.

51. Chen, X., Huang, L., Yang, Y., Chen, S., Sun, J., Ma, C., Xie, J., Song, Y., and Yang, J. (2020). ASPM promotes glioblastoma growth by regulating G1 restriction point progression and Wnt-β-catenin signaling. Aging *12*, 224–241.

52. Raisch, J., Cote-Biron, A., and Rivard, N. (2019). A Role for the WNT Co-Receptor LRP6 in Pathogenesis and Therapy of Epithelial Cancers. Cancers *11*, 1162. https://doi.org/10.3390/cancers11081162.

53. Zhang, J., Chen, J., Wo, D., Yan, H., Liu, P., Ma, E., Li, L., Zheng, L., Chen, D., Yu, Z., et al. (2019). LRP6 Ectodomain Prevents SDF-1/CXCR4-Induced Breast Cancer Metastasis to Lung. Clin. Cancer Res. *25*, 4832–4845. https://doi.org/10.1158/1078-0432.CCR-18-3557.

54. Guan, B., Ran, X.-G., Du, Y.-Q., Ren, F., Tian, Y., Wang, Y., and Chen, M.-M. (2018). High CHD9 expression is associated with poor prognosis in clear cell renal cell carcinoma. Int. J. Clin. Exp. Pathol. *11*, 3697–3702.

55. She, K., Yang, W., Li, M., Xiong, W., and Zhou, M. (2021). FAIM2 Promotes Non-Small Cell Lung Cancer Cell Growth and Bone Metastasis by Activating the Wnt/beta-Catenin Pathway. Front. Oncol. *11*, 690142. https://doi.org/10.3389/fonc.2021.690142.

56. Röst, H.L., Rosenberger, G., Navarro, P., Gillet, L., Miladinović, S.M., Schubert, O.T., Wolski, W., Collins, B.C., Malmström, J., Malmström, L., and Aebersold, R. (2014). OpenSWATH enables automated, targeted analysis of data-independent acquisition MS data. Nat. Biotechnol. *32*, 219–223. https://doi.org/10.1038/nbt.2841.

57. Demichev, V., Messner, C.B., Vernardis, S.I., Lilley, K.S., and Ralser, M. (2020). DIA-NN: neural networks and interference correction enable deep proteome coverage in high throughput. Nat. Methods *17*, 41–44. https://doi.org/10.1038/s41592-019-0638-x.

58. MacLean, B., Tomazela, D.M., Shulman, N., Chambers, M., Finney, G.L., Frewen, B., Kern, R., Tabb, D.L., Liebler, D.C., and MacCoss, M.J. (2010). Skyline: an open source document editor for creating and analyzing targeted proteomics experiments. Bioinformatics *26*, 966–968. https://doi.org/10.1093/bioinformatics/btq054.

59. Martinez-Val, A., Bekker-Jensen, D.B., Hogrebe, A., and Olsen, J.V. (2021). Data Processing and Analysis for DIA-Based Phosphoproteomics Using Spectronaut, Proteomics Data Analysis. Methods Mol. Biol. *2361*, 95–107. https://doi.org/10.1007/978-1-0716-1641-3_6.

60. Ma, J., Chen, T., Wu, S., Yang, C., Bai, M., Shu, K., Li, K., Zhang, G., Jin, Z., He, F., et al. (2019). iProX: an integrated proteome resource. Nucleic Acids Res. *47*, D1211–D1217. https://doi.org/10.1093/nar/gky869.

61. Chambers, M.C., Maclean, B., Burke, R., Amodei, D., Ruderman, D.L., Neumann, S., Gatto, L., Fischer, B., Pratt, B., Egertson, J., et al. (2012). A cross-platform toolkit for mass spectrometry and proteomics. Nat. Biotechnol. *30*, 918–920. https://doi.org/10.1038/nbt.2377.

Patterns, Volume *4*

Supplemental information

DPHL v.2: An updated and comprehensive DIA pan-human assay library for quantifying more than 14,000 proteins

Zhangzhi Xue, Tiansheng Zhu, Fangfei Zhang, Cheng Zhang, Nan Xiang, Liujia Qian, Xiao Yi, Yaoting Sun, Wei Liu, Xue Cai, Linyan Wang, Xizhe Dai, Liang Yue, Lu Li, Thang V. Pham, Sander R. Piersma, Qi Xiao, Meng Luo, Cong Lu, Jiang Zhu, Yongfu Zhao, Guangzhi Wang, Junhong Xiao, Tong Liu, Zhiyu Liu, Yi He, Qijun Wu, Tingting Gong, Jianqin Zhu, Zhiguo Zheng, Juan Ye, Yan Li, Connie R. Jimenez, Jun A, and Tiannan Guo

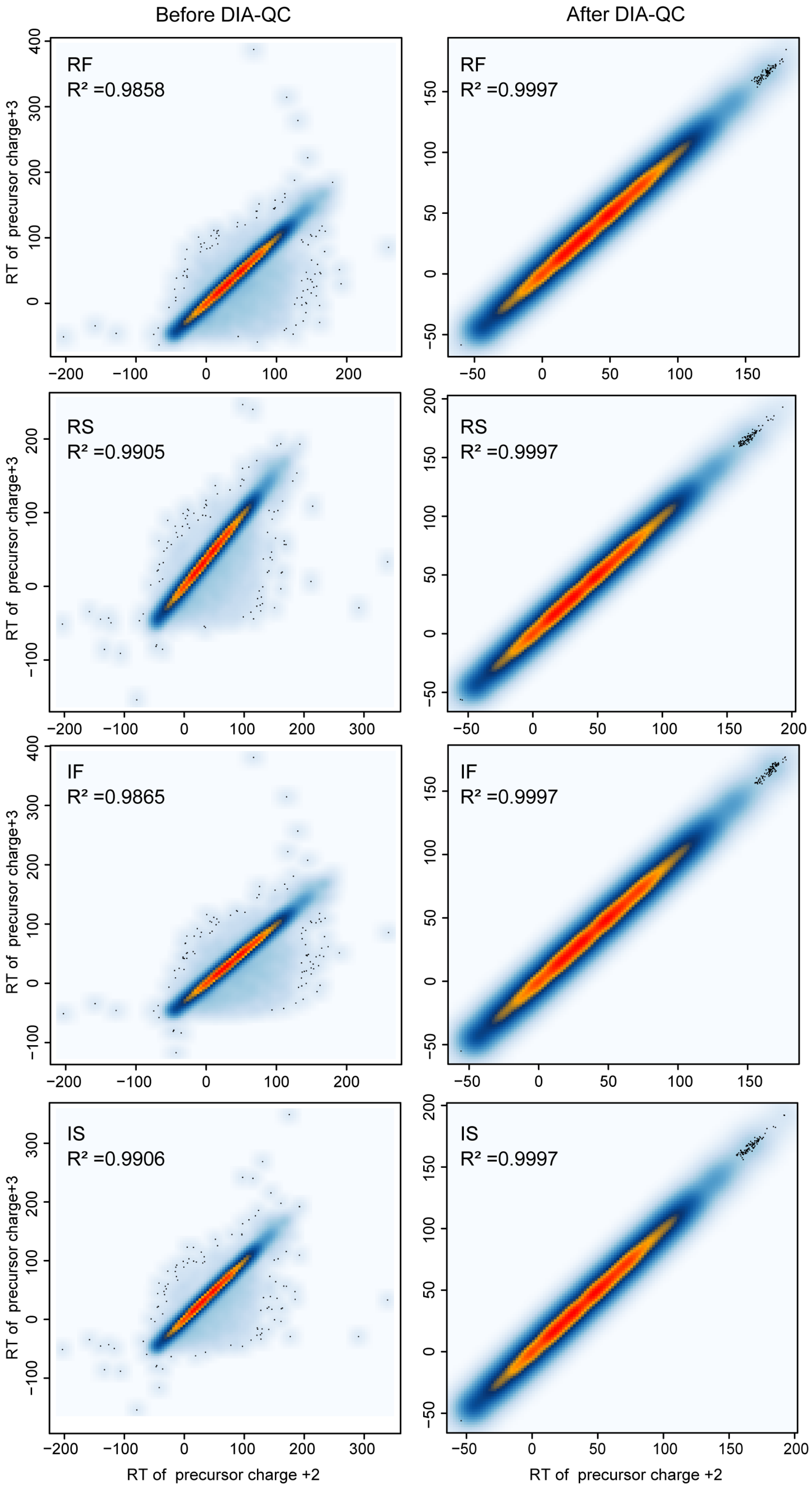

Figure S1. Correlation of paired peptides with RT correction values before and after QC.

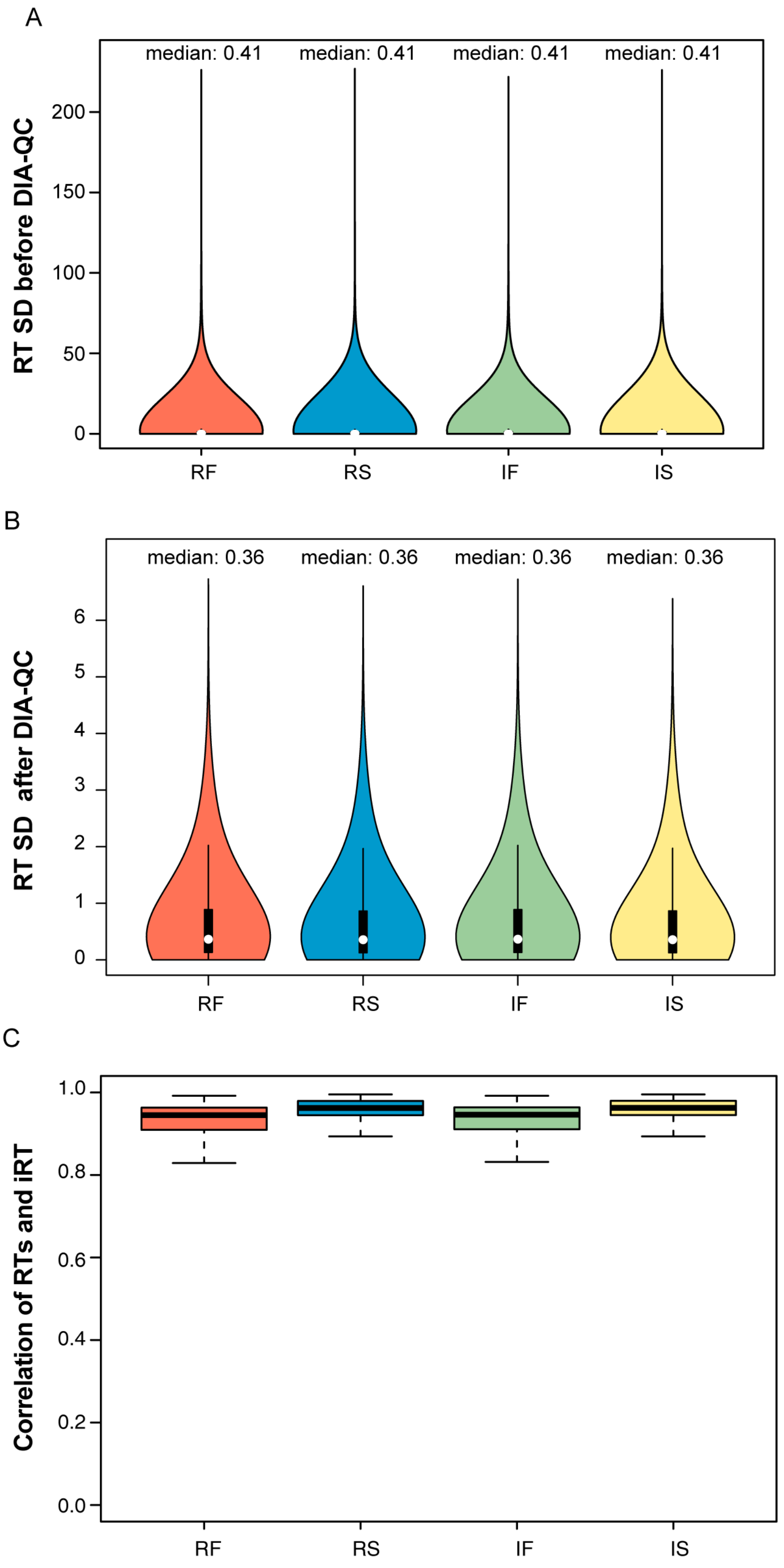

Figure S2. Standard deviation (SD) of paired peptides with RT values before (A) and after (B) QC. (C) The correlation between the RTs and iRT values in each sample of the four libraries.

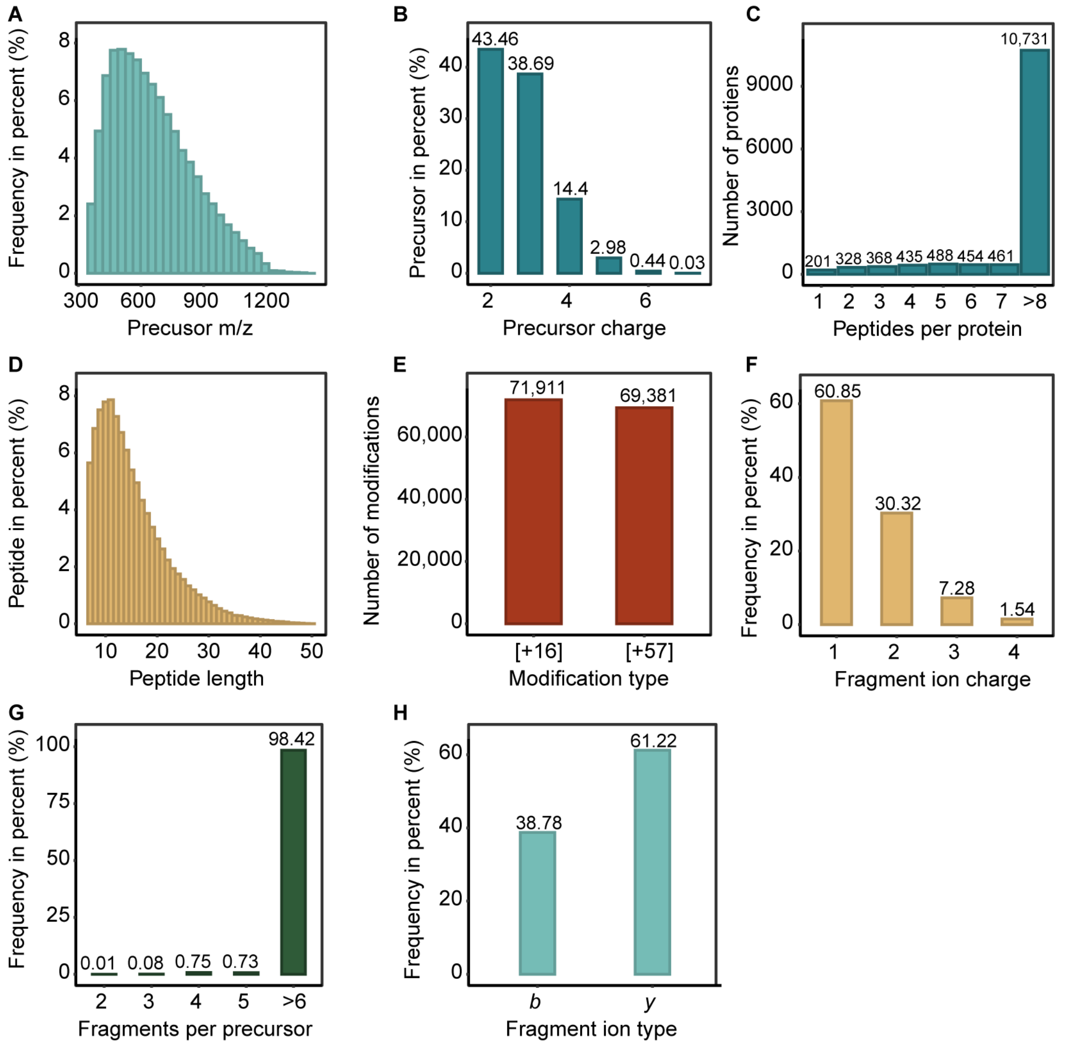

Figure S3. Characteristics of the RF library. (A) Distribution of precursors' m/z. (B) Counts of different precursor charge states. (C) Number of proteotypic peptides for each protein. (D) Distribution of peptide lengths. (E) Number of peptides with either of two modifications (+16: oxidation in methionine; +57: carbamidomethylation in cysteine). (F) The proportion of different charges of fragment ions. (G) Proportion of fragment ions per precursor ion. (H) Percentage of b and y ions.

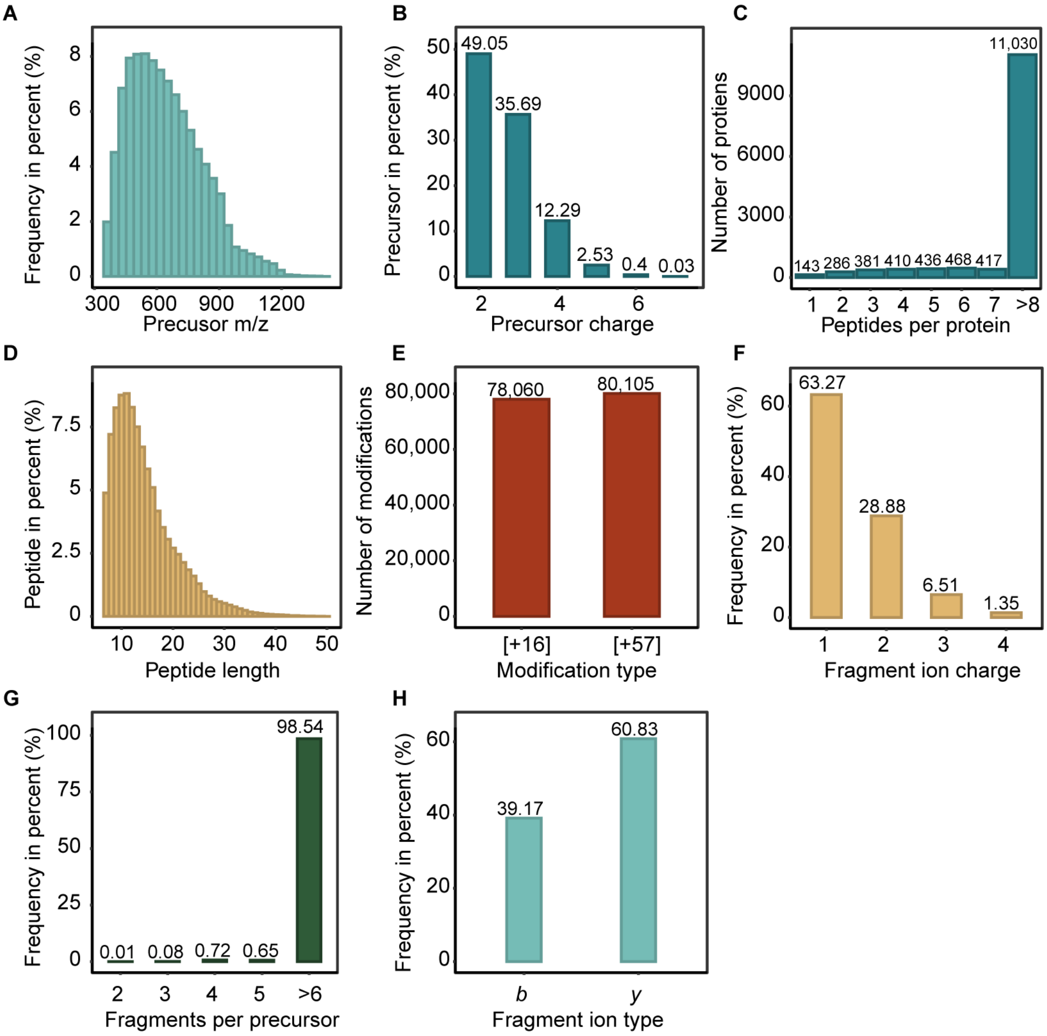

Figure S4. Characteristics of the RS library. (A) Distribution of precursors' m/z. (B) Counts of different precursor charge states. (C) Number of proteotypic peptides for each protein. (D) Distribution of peptide lengths. (E) Number of peptides with either of two modifications (+16: oxidation in methionine; +57: carbamidomethylation in cysteine). (F) The proportion of different charges of fragment ions. (G) Proportion of fragment ions per precursor ion. (H) Percentage of b and y ions.

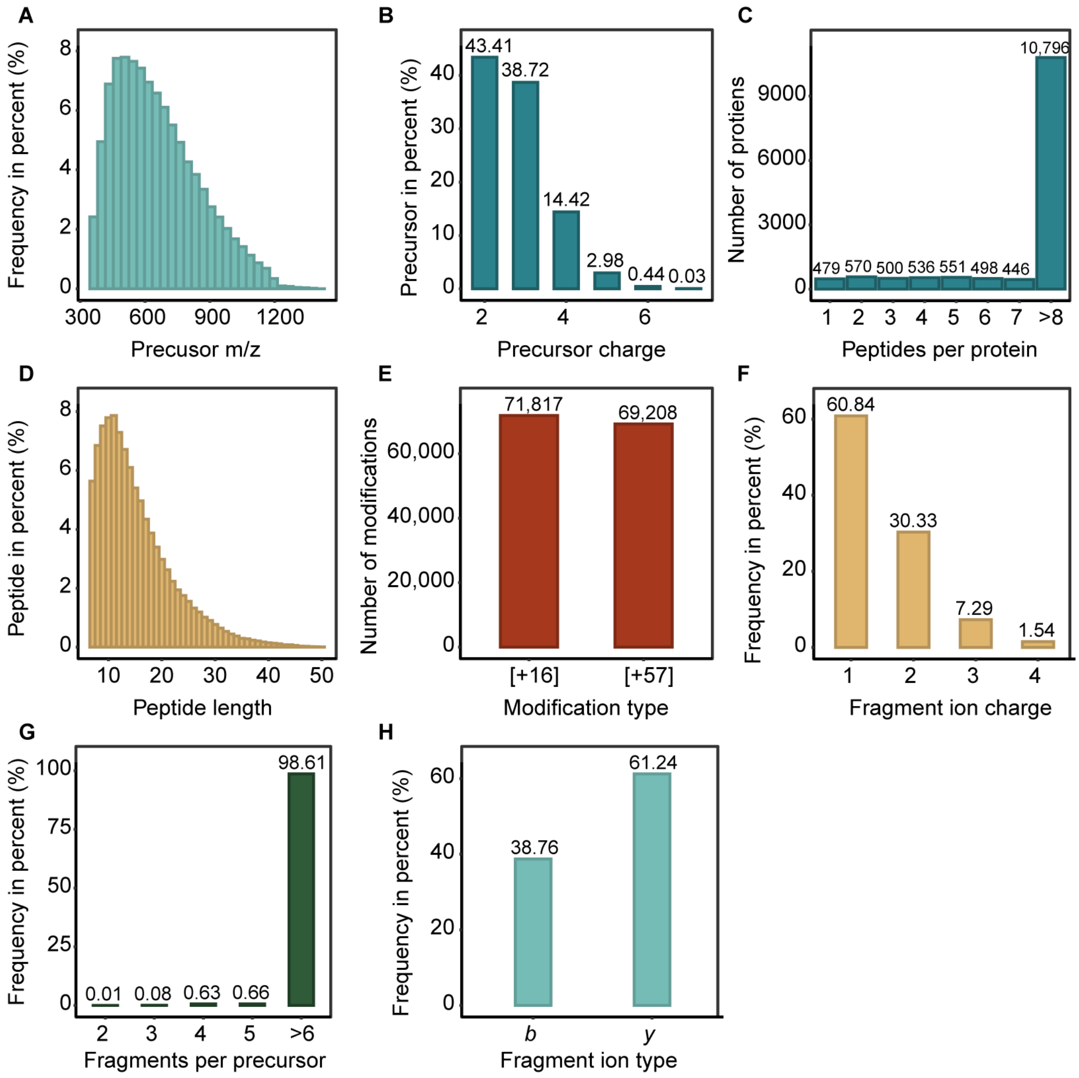

Figure S5. Characteristics of the IF library. (A) Distribution of precursors' m/z. (B) Counts of different precursor charge states. (C) Number of proteotypic peptides for each protein. (D) Distribution of peptide lengths. (E) Number of peptides with either of two modifications (+16: oxidation in methionine; +57: carbamidomethylation in cysteine). (F) The proportion of different charges of fragment ions. (G) Proportion of fragment ions per precursor ion. (H) Percentage of b and y ions.

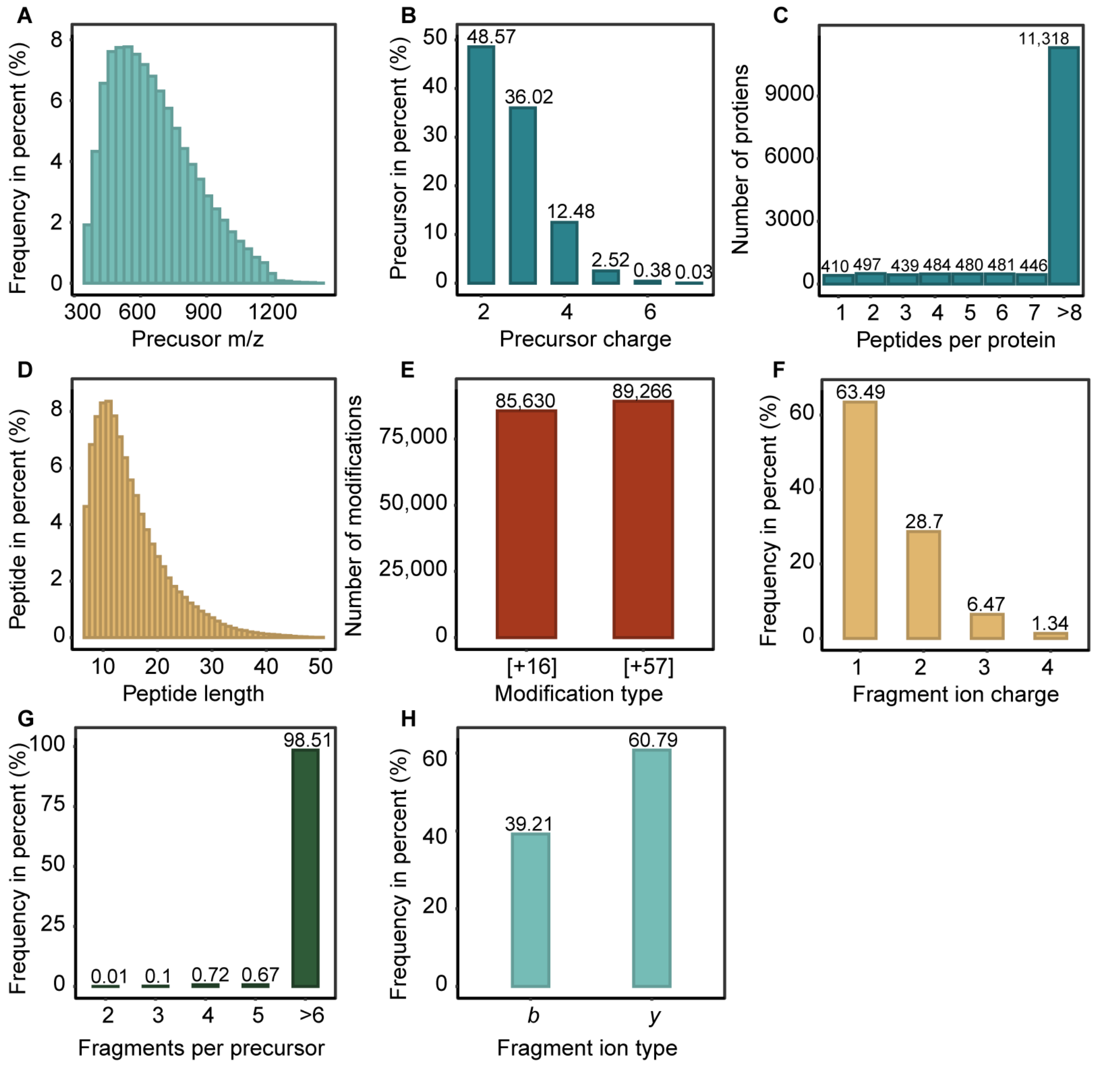

Figure S6. Characteristics of the IS library. (A) Distribution of precursors' m/z. (B) Counts of different precursor charge states. (C) Number of proteotypic peptides for each protein. (D) Distribution of peptide lengths. (E) Number of peptides with either of two modifications (+16: oxidation in methionine; +57: carbamidomethylation in cysteine). (F) The proportion of different charges of fragment ions. (G) Proportion of fragment ions per precursor ion. (H) Percentage of b and y ions.

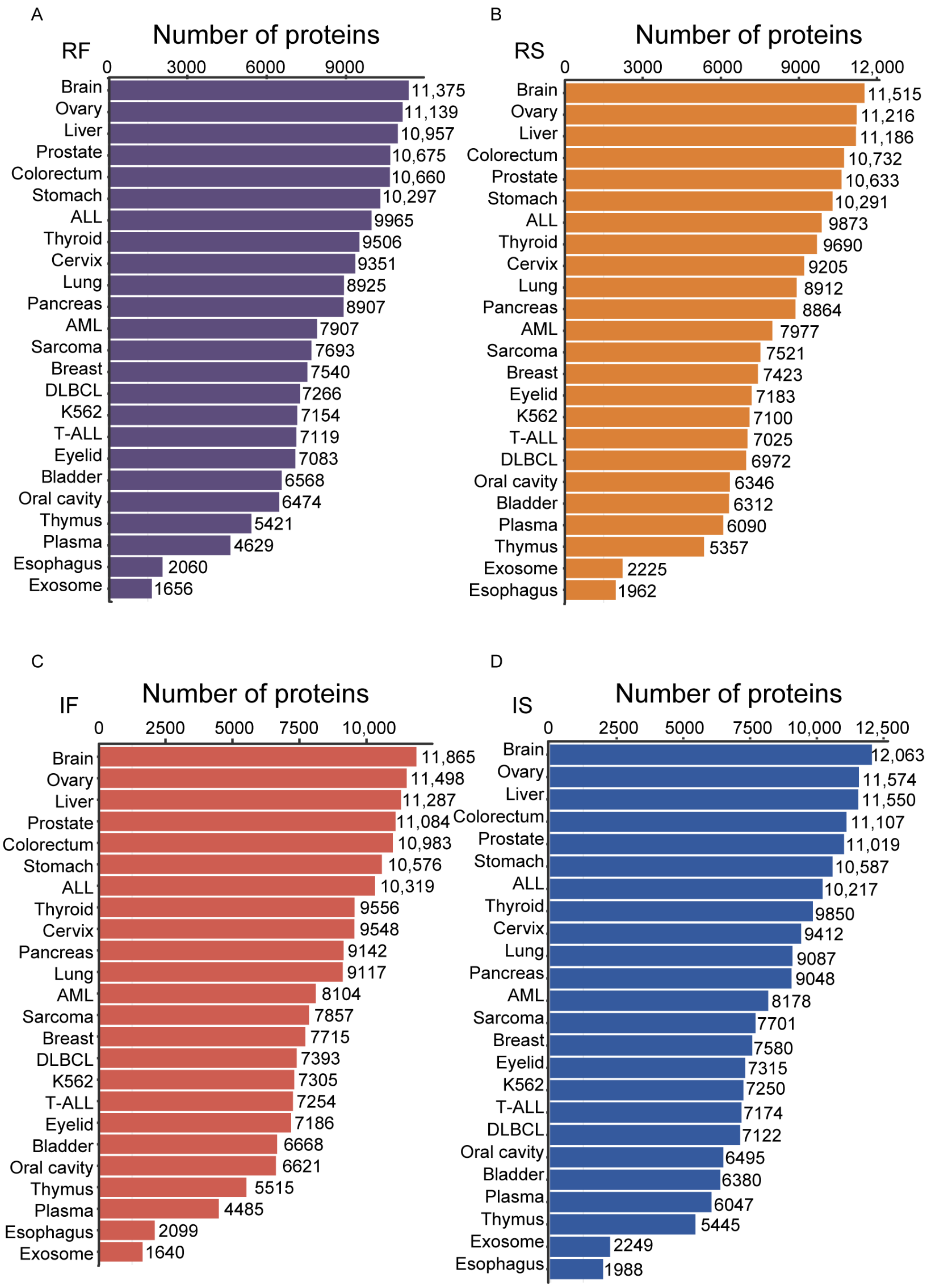

Figure S7. Bar plots displaying the number of identified proteins using our four libraries for each sample type. RF, reviewed fasta sequence & full-specific digestion mode; RS, reviewed fasta sequence & semi-specific digestion mode; IF, isoform fasta sequence & full-specific digestion mode; IS, isoform fasta sequence & semi-specific digestion mode; ALL, acute lymphoblastic leukemia.

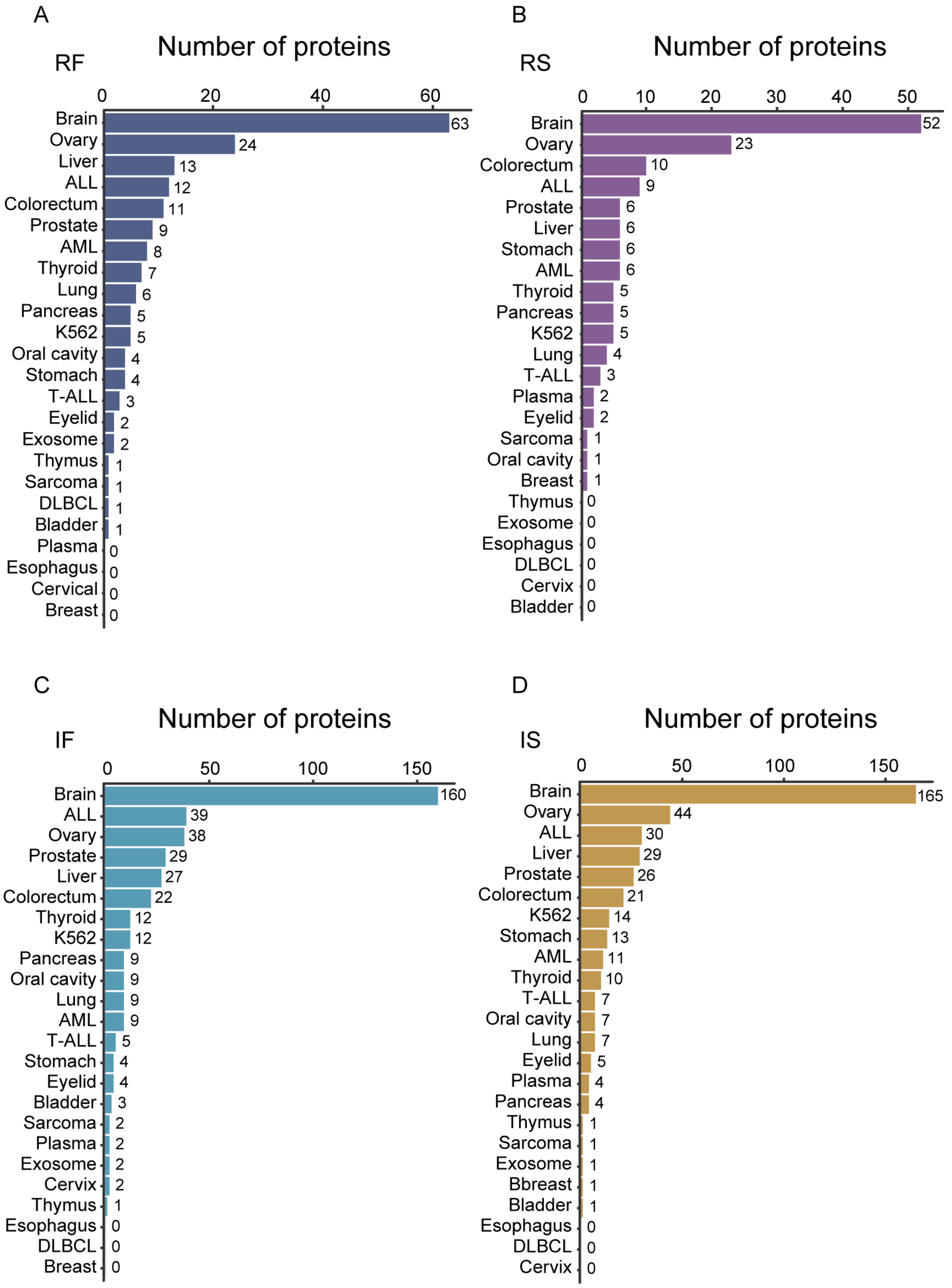

Figure S8. Bar plots displaying the number of identified unique proteins for each sample type. RF, reviewed fasta sequence & full-specific digestion mode; RS, reviewed fasta sequence & semi-specific digestion mode; IF, isoform fasta sequence & full-specific digestion mode; IS, isoform fasta sequence & semi-specific digestion mode; ALL, acute lymphoblastic leukemia.

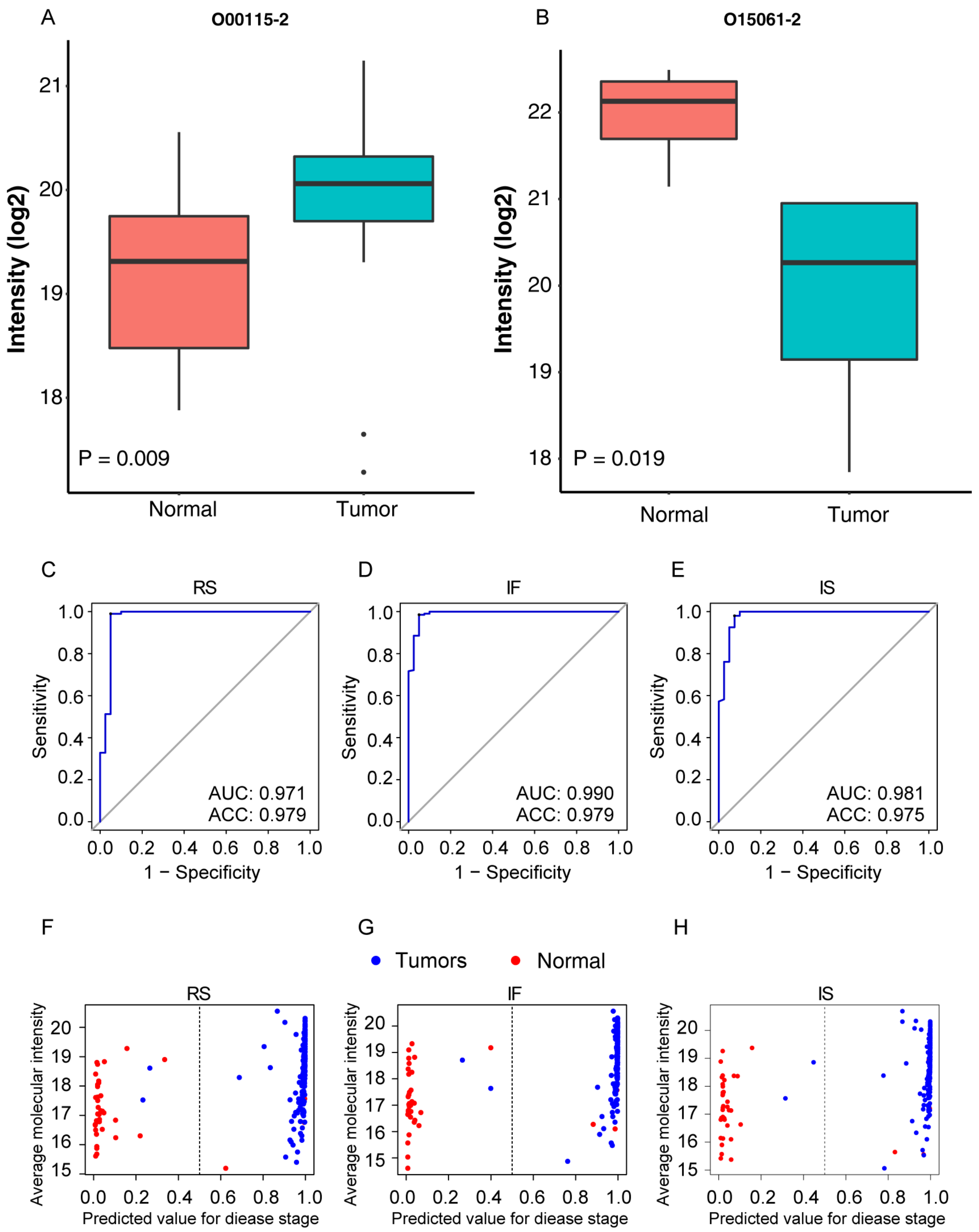

Figure S9. (A-B) The expression levels of the O00115-2 and the O15061-2. (C-E) ROC plots of the RS set, the IF set, and the IS set. (F-H) Performance of the model in the RS set, the IF set, and the IS set. RF, reviewed fasta sequence & full-specific digestion mode; RS, reviewed fasta sequence & semi-specific digestion mode; IF, isoform fasta sequence & full-specific digestion mode; IS, isoform fasta sequence & semi-specific digestion mode.

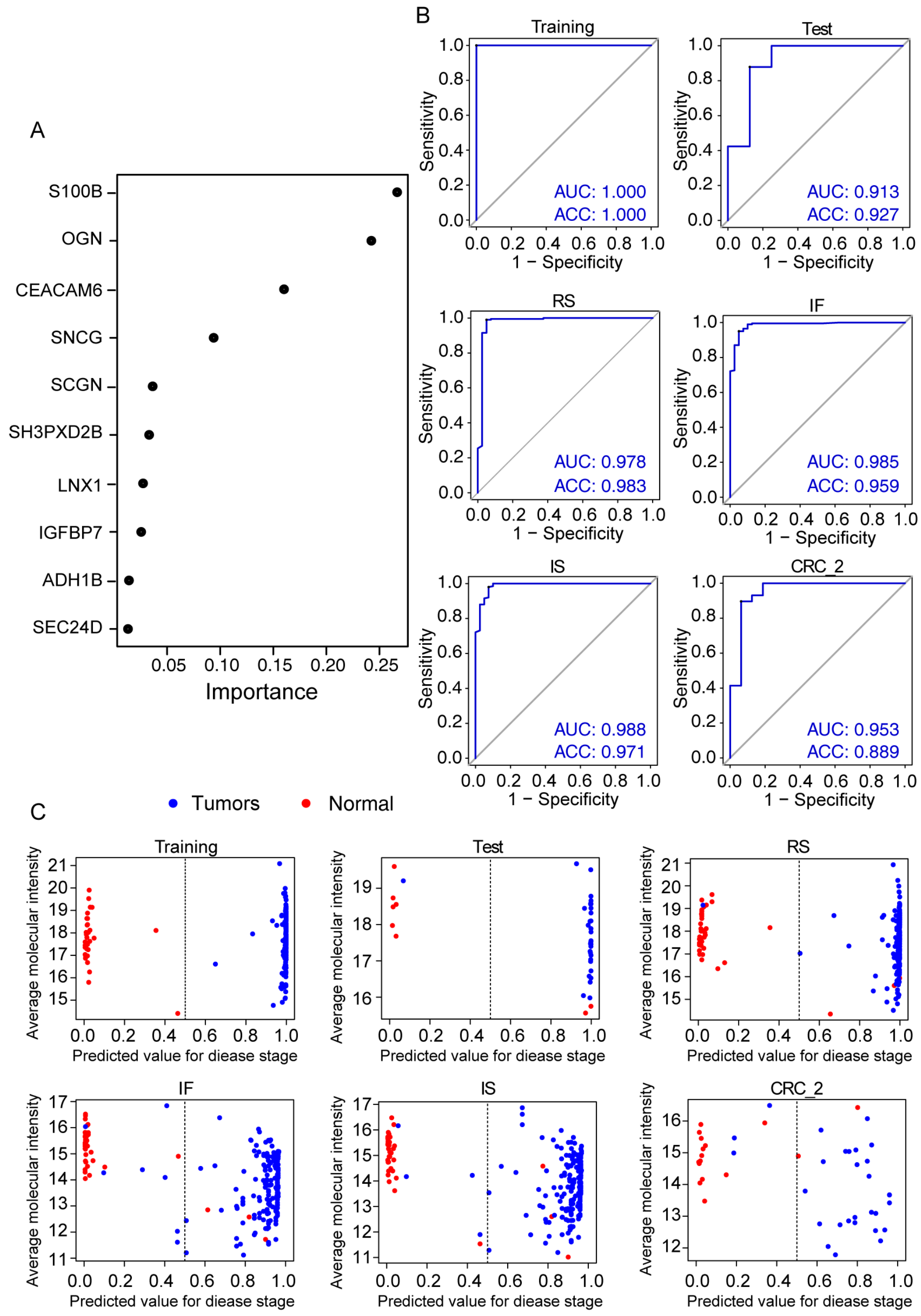

Figure S10. Machine learning to identify potential CRC biomarkers (using all differentially expressed proteins from the RF set). (A) Prioritization of 10 important variables. (B) Performance of the model in the training set, the test set, the RS set, the IF set, the IS set, and the CRC_2 dataset. RF, reviewed fasta sequence & full-specific digestion mode; RS, reviewed fasta sequence & semi-specific digestion mode; IF, isoform fasta sequence & full-specific digestion mode; IS, isoform fasta sequence & semi-specific digestion mode.
